# Structure-based function analysis of putative conserved proteins with isomerase activity from *Haemophilus influenzae*

**DOI:** 10.1007/s13205-014-0274-1

**Published:** 2014-12-28

**Authors:** Mohd. Shahbaaz, Faizan Ahmad, Md. Imtaiyaz Hassan

**Affiliations:** 1 Department of Computer Science, Jamia Millia Islamia, Jamia Nagar, New Delhi, 110025 India; 2 Center for Interdisciplinary Research in Basic Sciences, Jamia Millia Islamia, Jamia Nagar, New Delhi, 110025 India

**Keywords:** *Haemophilus influenza*, Hypothetical protein, Isomerase activity, Structure prediction, Structure analysis, Structure–function relationship

## Abstract

*Haemophilus influenzae*, a Gram-negative bacterium and a member of the family *Pasteurellaceae,* causes chronic bronchitis, bacteremia, meningitis, etc. The *H. influenzae* is the first organism whose genome was completely sequenced and annotated. Here, we have extensively analyzed the genome of *H. influenzae* using available proteins structure and function analysis tools. The objective of this analysis is to assign a precise function to hypothetical proteins (HPs) whose functions are not determined so far. Function prediction of these proteins is helpful in precise understanding of mechanisms of pathogenesis and biochemical pathways important for selecting novel therapeutic target. After an extensive analysis of *H. Influenzae* genome we have found 13 HPs showing high level of sequence and structural similarity to the enzyme isomerase. Consequently, the structures of HPs have been modeled and analyzed to determine their precise functions. We found these HPs are alanine racemase, lysine 2, 3-aminomutase, topoisomerase DNA-binding C4 zinc finger, pseudouridine synthase B, C and E (Rlu B, C and E), hydroxypyruvate isomerase, nucleoside-diphosphate-sugar epimerase, amidophosphoribosyltransferase, aldose-1-epimerase, tautomerase/MIF, Xylose isomerase-like, have TIM barrel domain and sedoheptulose-7-phosphate isomerase like activity, signifying their corresponding functions in the *H. influenzae*. This work provides a better understanding of the role HPs with isomerase activities in the survival and pathogenesis of *H. influenzae*.

**Electronic supplementary material:**

The online version of this article (doi:10.1007/s13205-014-0274-1) contains supplementary material, which is available to authorized users.

## Introduction

*Haemophilus influenzae,* a member of family *Pasteurellaceae,* is a non-motile Gram-negative bacterium (Kuhnert, 2008). It is an obligatory human parasite which causes meningitis, sinusitis, epiglottitis, chronic bronchitis and community acquired pneumonia (Apisarnthanarak and Mundy 2005; Eldika and Sethi 2006). The genome of *H. influenzae* was successfully sequenced (Fleischmann et al. 1995) which revealed 1,740 protein-coding genes, 2 transfer RNA genes, and 18 other RNA genes in a 1.83 Mb single circular chromosome (Fleischmann et al. 1995). *H. influenzae* requires β-nicotinamide adenine dinucleotide and heme-related compounds for its growth (Markel et al. 2007; Morton et al. 2004a). Hence, it uses numerous mechanisms to obtain heme (Stojiljkovic and Perkins-Balding 2002) using various heme acquisition proteins like Hup protein (Morton et al. 2004b) and HbpA lipoprotein (Morton et al. 2005). It is also evident that the periplasmic iron-binding protein, FbpA (ferric-ion-binding protein A), plays an essential role in procurement of iron from transferrin in *H. influenzae* (Khun et al. 1998; Kirby et al. 1997). This shows that iron is important for its survival and virulence (Morton et al. 2004a). Furthermore, there is a strict regulation of iron homeostasis in *H. influenzae* as indicated by the mechanism for heme acquisition in the organism.

*H. influenzae* strains comprise high antibiotic resistance, including multidrug resistance to ampicillin and chloramphenicol, make the treatment of meningitis and chronic pneumonia more complex (Campos 2001; Pfeifer et al. 2013; Saha et al. 2008). The antibiotic resistance in *H. influenzae* was strongly associated with the presence of large conjugative plasmids (Leaves et al. 2000). The antibiotic resistances in *H. influenzae* occur due to various mechanisms which can affect the empirical treatment of infections (Jorgensen, 1991; Kostyanev and Sechanova, 2012; Tristram et al. 2007). There is an increasing prevalence of resistance to antibiotics like aminopenicillins, macrolides, tetracyclines and fluoroquinolones. This is a major associated problem (Jorgensen, 1991; Kostyanev and Sechanova, 2012; Tristram et al. 2007). An extensive genome analysis of the organism may be helpful to find novel drug targets against multidrug-resistant strains.

Analysis of 102 bacterial genomes of the genomic consortium reflects that 45,110 proteins are prearranged in 7,853 orthologous groups with unknown function (Doerks et al. 2004). These proteins are considered as a “conserved hypothetical proteins (HPs)”, i.e., proteins that have not been functionally characterized and described at biochemical and physiological level in organisms (Galperin and Koonin 2004). The HPs are supposed to be the products of pseudogenes in majority of organisms and comprise a wide fraction of their proteomes (Desler et al. 2012; Galperin 2001). The species-specific phenotypic properties such as pathogenicity in a given organism can be determined by analyzing unique sequences of HPs because these determinants are assumed to be the potent drug targets in pathogenic strains of organisms (Tsoka and Ouzounis 2000). The significance of functional characterization HP can further be understood by recent functional annotation of formerly uncharacterized tRNA modification enzymes (Alexandrov et al. 2002; Jackman et al. 2003; Soma et al. 2003) of the deoxyxylulose pathway (Eisenreich et al. 2001) that plays a central role in cyclic diguanylate bacterial signaling (Galperin 2004; Jenal 2004). We have been working in the area of structure-based rational drug design hence we are searching a novel therapeutic target in pathogenic organism (Hassan et al. 2007a, b; Thakur et al. 2013a). We have successfully annotated the function of HPs from pathogenic organism both at sequence and structure levels (Kumar et al. 2014a, b; Shahbaaz et al. 2014; Sinha et al. 2014).

The biological function cannot be predicted by comparison of sequence similarity alone (Illergard et al. 2009). Structure-based function prediction is often considered as a better tool in comparison to the sequence-based methods. Because in most cases the evolution retains a conserved folding pattern despite of very poor sequence similarity (Hassan and Ahmad 2011; Hassan et al. 2008, 2013; Illergard et al. 2009). Furthermore, identification of binding motifs and catalytic sites is critical for a protein function, which can easily be predicted from the available protein structure (Shapiro and Harris 2000; Singh et al. 2014). Moreover, the process of structure-based rational drug design is completely based on the structural features of a protein molecule (Capdeville et al. 2002; Klebe 2000; Tasleem et al. 2014; Thakur et al. 2013b). Hence, structure analysis of HPs is central to strengthen the process of biological function prediction and development of better therapeutics intervention for the treatment of diseases associated with the pathogen.

Earlier, we have successfully predicted lyases from the same organism (Shahbaaz et al. 2014). Here, extensive sequence analysis of *H. influenzae*, we identified 13 HPs that possess isomerase-like activity, are listed in Table 1. The isomerase enzymes are directly associated with virulence (Reffuveille et al. 2012; Ren et al. 2005) because these enzymes provide a favorable local environment to pathogens in the host for their growth (Bjornson 1984). It was reported that the enzyme Ess1 prolyl isomerase plays an important role in the pathogenesis of fungi *Cryptococcus neoformans* (Ren et al. 2005). Isomerases play important role in the generation of resistance against β-lactam antibiotics (Reffuveille et al. 2012). Phosphomannose isomerase is involved in the *Leishmania* pathogenesis. All these evidences suggest that sequence and structure analysis of isomerase enzymes will be helpful for the better understanding of a precise function of these enzymes and will open a new promising target for structure-based rational drug design.

**Table 1:** List of HPs with isomerase activity from *H. influenzae* Rd KW

S.no	Accession no	Gene id	Protein product	Uniprot id	Protein name
1.	NC_000907.1	950992	NP_438263.1	P44506	HP HI0090
2.	NC_000907.1	949459	NP_438493.1	P44641	HP HI0329
3.	NC_000907.1	949423	NP_438817.1	P46494	HP HI0656.1
4.	NC_000907.1	950211	NP_438854.1	P44827	HP HI0694
5.	NC_000907.1	950733	NP_439174.1	Q57151	HP HI1013
6.	NC_000907.1	950006	NP_439175.1	P44094	HP HI1014
7.	NC_000907.1	950150	NP_439355.1	P45104	HP HI1199
8.	NC_000907.1	950157	NP_439364.1	P71373	HP HI1208
9.	NC_000907.1	950209	NP_439468.1	P44160	HP HI1317
10.	NC_000907.1	950703	NP_439541.1	O86237	HP HI1388.1
11.	NC_000907.1	950784	NP_439587.1	Q57152	HP HI1436
12.	NC_000907.1	950455	NP_439742.1	P44268	HP HI1600
13.	NC_000907.1	950796	NP_439799.1	P52606	HP HI1657

## Materials and methods

### Sequence retrieval

Extensive analysis of *H. influenzae* genome shows 1,657 proteins which are encoded by its genome (http://www.ncbi.nlm.nih.gov/genome/?term=haemophilus+influenzae). We have already characterized 429 proteins as HP in *H. influenzae* and their FASTA sequences were retrieved from UniProt (http://www.uniprot.org/) using the “Gene ID” (Shahbaaz et al. 2013). After sequence analysis, we classified all 429 HPs into various classes using the information available in the publically available databases like PDB, Pfam, etc.(Shahbaaz et al. 2013). Here, we selected HPs with isomerase activity for further structure analysis. All tools used in this study are listed in the Table S1.

### Sequence analysis

We used several bioinformatics tools such as PSORTb (Yu et al. 2010b), PSLpred (Bhasin et al. 2005) and CELLO (Yu et al. 2006) to identify the subcellular localization of HPs. Furthermore, we also analyzed the presence of signal peptide using SignalP 4.1 (Emanuelsson et al. 2007) and to identify non-classical secretory pathway protein we used SecretomeP (Bendtsen et al. 2005). To characterize a protein to be a membrane protein, the online servers TMHMM (Krogh et al. 2001) and HMMTOP (Tusnady and Simon 2001) were used. Conserved sequence patterns in protein families were used for the prediction of the functions of HPs (Chen and Jeong 2000). The BLASTp (Altschul et al. 1990) and HHpred (Soding et al. 2005) were used for remote homology detection against various available protein databases such as PDB (Bernstein et al. 1978), SCOP (Hubbard et al. 1999) and CATH (Sillitoe et al. 2013). We further performed domain analysis of proteins for more precise function prediction of HPs [47]. The databases such as Pfam (Punta et al. 2011), PANTHER (Mi et al. 2005), SMART (Letunic et al. 2012), SUPERFAMILY (Gough et al. 2001), CATH (Sillitoe et al. 2012), CDART (Geer et al. 2002), SYSTERS (Meinel et al. 2005), ProtoNet (Rappoport et al. 2011) and SVMProt (Cai et al. 2003) were used for precise domain annotation in HPs. Similarly, instead of direct sequence similarity, we also used domain architecture and profile-based methods like CDART and SMART for similarity search.

The annotation of signature protein sequences was performed using the program MOTIF (Kanehisa 1997) and InterProScan (Quevillon et al. 2005). For the identification of motif sequence, we used MEME suite (Bailey et al. 2009). In addition, we also performed virulence factor prediction using VICMpred (Saha and Raghava 2006) and Virulentpred (Garg and Gupta 2008), since virulence factors are considered as potential drug/vaccine targets (Baron and Coombes 2007). We also acknowledge the importance of understanding the protein function using the information of protein–protein interactions. Therefore, to predict the interaction partners of HPs we used STRING (version–9.05) (Szklarczyk et al. 2011a, 2011b).

### Structure prediction

For modeling three-dimensional structure of HPs, we used two classes of structure prediction methodologies (Baker and Sali, 2001) (i.e., threading/comparative modeling and de novo or ab initio methods). The MODELLER (Eswar et al. 2006) module of Discovery Studio 3.5 (Accelrys 2013), I-TASSER (Roy et al. 2010) and ROBETTA server (Kim et al. 2004) were used for prediction of a reliable structure of HPs. We used the homology modeling (Marti-Renom et al. 2000) for structure prediction of those HPs where the sequence identity is >30 % between the target and template sequences. We, first, identified templates using sequence similarity search methods like PSI-BLAST (Altschul et al. 1997) present in Discovery Studio 3.5 (Accelrys 2013) for identification of potential templates in protein data bank (PDB). We also used the fold recognition methods like HHpred (Soding et al. 2005) for template identification. The template and query sequences were aligned and used for modeling HPs structures in MODELLER (Eswar et al. 2006).

In case of sequence identity <30 %, we used ab initio modeling protocols for predicting the structure. The I-TASSER (Roy et al. 2010) server uses ab initio algorithms, first generates three-dimensional (3D) atomic models from multiple threading alignments and iterative structural assembly simulations. It inferred function of the HPs using the structural matching of the 3D models with other known proteins and produced outputs contain full-length tertiary as well as secondary structure predictions, ligand-binding sites, Enzyme Commission (EC) numbers, etc. (Roy et al. 2010).

Similarly, ROBETTA server (Kim et al. 2004) also uses ab initio or de novo methods to predict the structure of proteins whose structural analogs do not exist in the PDB. First, it uses the alignment method, called K*Sync, to align the query sequence onto the parent structure. Then it models variable regions by allowing them to explore conformational space with fragments in a fashion similar to the de novo protocol in context of the template. Second, when no structural homolog is accessible, server modeled the domains using Rosetta de novo protocol (Misura et al. 2006), which allows the full length of the domain to explore conformational space via fragment inclusion, generating a sizeable decoy collection from which the concluding models are chosen.

The resulting models are optimized and then energy minimization was carried out using CHARM-22 from Accelrys Discovery Studio 3.5 and the steepest descent algorithm of GROMOS from Deepview (Kaplan and Littlejohn 2001). We further refined the predicted models of HPs using a side chain refinement protocol of Discovery studio 3.5 using force fields, like CHARMM (Brooks et al. 2009), and backbone-dependent rotamer library of SCWRL4 (Krivov et al. 2009) predicts positions of the side chains which are used for refinement of predicted protein structures. The loop refinement protocol of MODELLER (Eswar et al. 2006) is also used for improving the quality of predicted models.

### Structure validation

The quality of predicted HPs models were analyzed on SAVES server (Structural Analysis and Verification Server). The modeled protein structures are validated using PROCHECK (Laskowski et al. 1996), WHAT_CHECK (Hooft et al. 1996; Vriend 1990), ERRAT (Colovos and Yeates 1993), VERIFY_3D (Eisenberg et al. 1997; Luthy et al. 1992) and PROVE (Pontius et al. 1996) services present in SAVES server. PROCHECK validated the stereo-chemical quality of a protein structure by analyzing the overall structure and residue-by-residue geometry of the protein. Similarly, WHAT_CHECK also analyzes the stereo-chemical parameters of the residues in HPs. The ERRAT server of UCLA (University of California, Los Angeles) verifies the structures HPs by performing the statistical analysis of the patterns of non-bonded atomic interactions. Further, VERIFY_3D provides a visual analysis of the quality of HPs structures by determining the compatibility of predicted model of HP with its own primary structure. The PyMOL (DeLano 2002), a molecular graphics system, is used for visualization of protein structure and for calculating the *r.m.s.* deviation between the target HP and the template.

### Structure analysis

Structure similarity is more consistent than sequence similarity (Taylor and Orengo 1989). Since the structures of homologous proteins are more conserved than their sequences (Chothia and Lesk 1986). We used varieties of protein structure analysis tools for the prediction of function of HPs. CASP (Critical Assessment of protein Structure Prediction) contains firestar (Lopez et al. 2011), COACH (Yang et al. 2013), COFACTOR (Roy et al. 2012), 3DLigandSite (Wass et al. 2010), TM-SITE (Yang et al. 2013) and S-SITE (Yang et al. 2013), which were used for predicting catalytic and ligand-binding residues in protein sequences. We also used information available in literature about the templates used in protein modeling to identify the catalytic residues in HPs. Furthermore, active pocket sites in the predicted structures of HPs were identified using POCASA (Yu et al. 2010) and Pocket-Finder (Laurie and Jackson, 2005) servers. The PPM server (Lomize et al. 2012) was used for calculating spatial positions in membranes of HPs. The ProFunc (Laskowski et al. 2005) web server was used for structure-based function annotation and for predicting structural motifs associated with catalytic functions. Function predictions of HPs are also complimented by DALI server that compares the target structure with known structure submitted in PDB. The secondary structure elements are computed from atomic resolution protein structures of HPs using the STRIDE web server (Heinig and Frishman 2004).

## Result and discussion

Here, we performed sequence and structure analysis of 13 HPs which was predicted to be isomerase such as alanine racemase, lysine 2, 3-aminomutase, topoisomerase DNA-binding C4 zinc finger, pseudouridine synthase B, C and E hydroxypyruvate isomerase, nucleoside-diphosphate-sugar epimerase, amidophosphoribosyl transferase, aldose-1-epimerase, tautomerase/MIF, xylose isomerase-like, TIM barrel domain, sedoheptulose-7-phosphate isomerase-like activity. We predicted the structures of all 13 HPs and analyzed them using available bioinformatics tools. The predicted models of P44506, P44641, P46494, P44827, Q57151, P44094, P45104, P71373, P44160, Q57152, P44268, P52606 show significant validation score on SAVES server. The outcomes of structural analysis for each protein are described here, separately.

### HP P44506

HP P44506 is localized in the cytoplasm and devoid of signal peptide and transmembrane helix (Table S2). Sequence analysis reveals that this HP is having alanine racemase activity (Table S3 and S4). The MEME suite discovered three sequence-based motifs in the HP namely, 151′-ENLPHLCLRGLM, 209′-PSAIKCGSTMV, 76′-EWHFIG (Table 2). The virulence factor analysis shows that HP P44506 is a virulent protein according to VirulentPred and a metabolism molecule according to VICMpred (Table S3). The functional protein association networks predicted by the String (Szklarczyk et al. 2011a, b) indicates that HP P44506 shows close interaction with holliday junction resolvase-like protein, pyrroline-5-carboxylate reductase, coproporphyrinogen III oxidase, cell division protein FtsZ, putative deoxyribonucleotide triphosphate pyrophosphatase, homoserine O-acetyltransferase, phosphatase and cell division protein according to STRING analysis (Szklarczyk et al. 2011a, b).

**Table 2:** List of sequence-based predicted function of HPs with isomerase activity and Motif discovered using MEME of *H. influenzae* strain Rd KW20

S.NO	Cluster^#^	UNIPROT ID	MEME results							Consensus^a^ function
			Motif 1		Motif 2		Motif 3		MAST function prediction	
			Start	Site	Start	Site	Start	Site		
1.	Cluster 102	P44506	151	ENLPHLCLRGLM	209	PSAIKCGSTMV	76	EWHFIG	UPF0001 protein	Alanine racemase
2.	Cluster 170	P44641	120	GCAVNC	236	IFAHAM	88	GFSTDP	l-lysine 2,3-aminomutase	lysine 2,3-aminomutase
3.	Cluster 152	P46494	76	FGMFIGCSHYPECDFVV	1	MNQSLFHH	115	RRGRQGKIFY	No result	Topoisomerase DNA-binding C4 zinc finger
4.	Cluster 80	P44827	84	VYAAGRLDRDSEGLLILTNNGELQHRLADPKFKTEKTYWVQVEGI	51	TKVVLFNKPFDVLTQFTDEQGRATLKD	178	WLEIKISEGRNRQVRRMTAHIGFP	Ribosomal large subunit pseudouridine synthase E	ribosomal large subunit pseudouridine synthase E
5.	Cluster 128	Q57151	99	CPNVHIM	71	WGGSAI	178	DYFHAQ	Putative hydroxypyruvate isomerase	hydroxypyruvate isomerase
6.	Cluster 162	P44094	149	MCELLINDYSRKGFVDGIVVRLPTICIRPGKPNKAASSFVSSIMREPLHG	55	CPVSEE	291	QALALGFKV	No result	Nucleoside-diphosphate-sugar epimerase
7.	Cluster 80	P45104	176	WIAVGRLDINTSGLLLFTTDGELANRLMHPSREVEREYSVRVFGQ	140	CRVLMYYKPEGELCTRSDPEGRATVFD	256	WYDVTLMEGRNREVRRLWESQGIQ	Ribosomal large subunit pseudouridine synthase B	ribosomal large subunit pseudouridine synthase B
8.	Cluster 113	P71373	209	DHSECRGAFNFAAPKSIKQH	284	DCENYL	268	VVPEKLLNAGFQFQY	Epimerase family protein HI_1208	Amidophosphoribosyltransferase (Epimerase)
9.	Cluster 38	P44160	86	QPAHGT	75	PICYPW	29	CGWNTKNFPC	Putative glucose-6-phosphate 1-epimerase	Aldose 1-epimerase
10.	Cluster 38	O86237	104	QPAHCW	48	DFYYPF	34	KGKHAIRFLC	No result	Tautomerase/MIF
11.	Cluster 114	Q57152	51	WVFIPRM	72	AISPYI	38	FSIDTM	No result	RNA pseudouridine synthase C
12.	Cluster 196	P44268	249	KGTVWD	99	CECEGH	35	ENWSKM	No result	Xylose isomerase-like, TIM barrel domain
13.	Cluster 141	P52606	97	ELYCHQ	32	QMVMQC	1	MLQKVK	No result	Sedoheptulose 7-phosphate isomerase

^a^Consensus result form on the basis of values present in Table S3 and S4

The sequence of HP P44506 was also annotated in the Unirpot database. We found that pyridoxal 5′-phosphate (PLP) is a cofactor for this protein, clearly indicated its role in the pyridoxal 5′-phosphate binding. It is interesting to note that sequence similarities searches showed that HP P44506 belongs to the uncharacterised protein family UPF0001, which is primarily involved in the biosynthesis of amino acids and amino acid-derived metabolites. Finally, family and domain database search analysis clearly indicates that HP P44506 containing N-terminal alanine racemase domain, PLP-binding barrel, belongs to racemases and epimerases and actis on amino acids and derivatives.

**Fig. 1:**
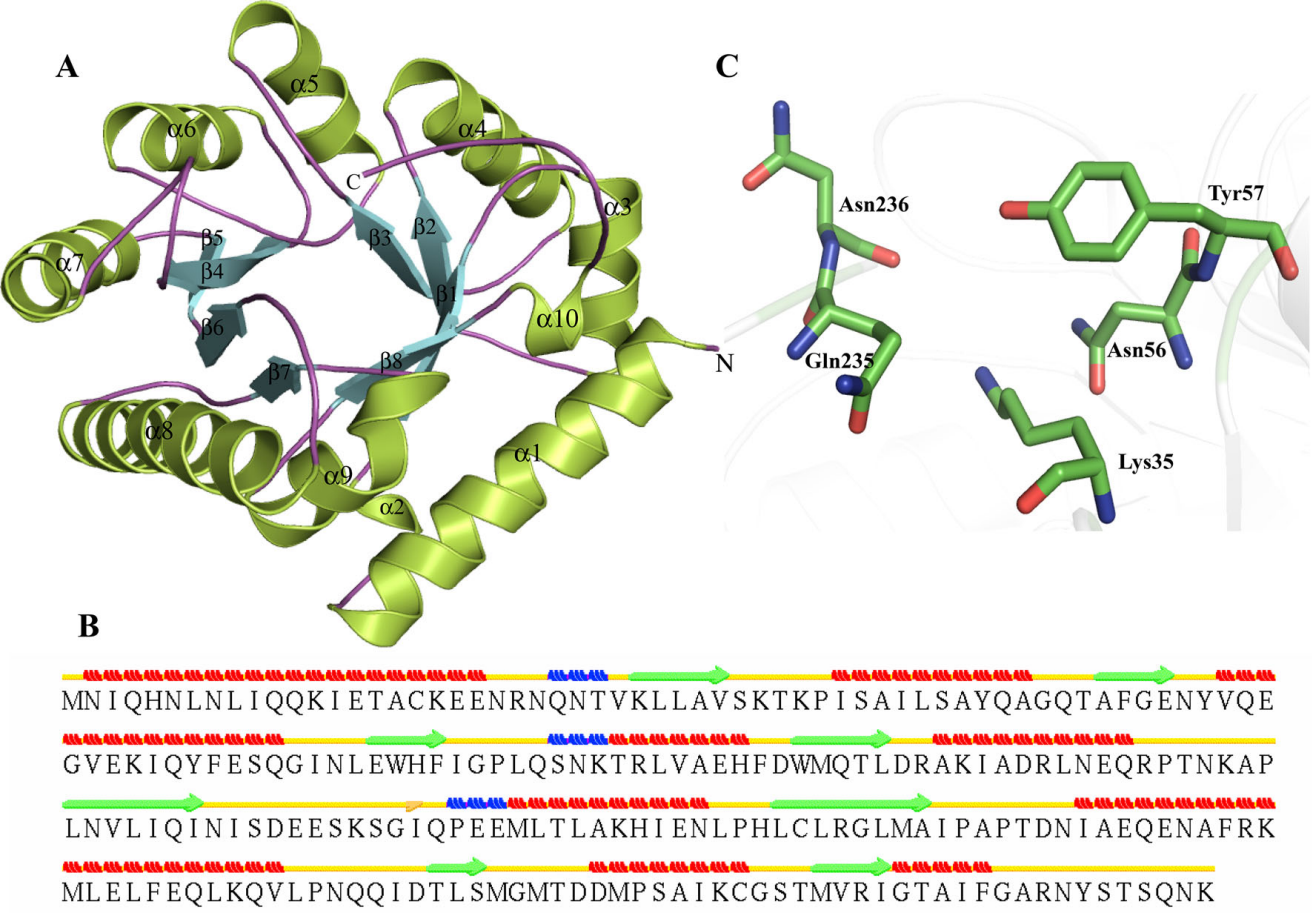
Representation of model structure of HP P44506. **a** Showing characteristic TIM barrel domain. **b** Secondary structure prediction of HPs using their three-dimensional structural framework by STRIDE, where α-helix, β-strands, loops, 3_10_ helix and β-bridges are represented *in red*, *green*, *yellow*, *blue* and *pink*, respectively (this illustration is applicable for all figures). **c** Residues present in the active site pocket are illustrated in *stick*

Three-dimensional structure of P44506 was predicted by MODELLER. HP P44506, which shows a sequence homology of 61 and 57 % with templates, PLP-binding protein (PDB ID—1W8G) and pyridoxal phosphate-binding protein (PDB ID—3SY1), respectively. The energy of minimized structure was validated showing 99.5 % of residues in the allowed region of the Ramachandran plot (Ramachandran et al. 1963) (Table 3). The root mean square deviation (RMSD) of the predicted model with that of templates 1W8G, 3SY1 and 4A3Q was 0.223 Å^2^, 0.243 Å^2^ and 3.997 Å^2^, respectively (Table 3), indicating a close functionality. The TM score of HP model with 1W8G, 3SY1 and 4A3Q is 0.6229, 0.5130 and 0.2651, respectively, showing that 1W8G and 3SY1 belong to the fold which is similar to that of P44506 (Table 3). Structure comparison and analysis revealed that P44506 contains (α/β)_8_ TIM barrel at the N terminus (Fig. 1a), a characteristics of carrying a phosphate-binding site. The overall structure of P44506 contains ten α-helices, three 3_10_ helices and eight β-strands forming the characteristic TIM barrel. This prediction is complimented using various binding site prediction servers. The structure also shows the presence of isolated β bridge at ILE138 (Fig. 1b). The P44506 TIM barrel domain contains eight β-strands (β1–β8) with characteristic PLP (pyridoxal-5-phosphate) binding site at Lys35 identified by structure similarity with the templates (Table S5). Further, Pocket-Finder analysis shows that the active site cavity may contain Lys35, Asn56, Tyr57, Gln235 and Asn236 (Fig. 1c).

**Table 3:** List of structure-based predicted function and validation of HP with isomerase activity in *H. influenzae* strain Rd KW20

S. no.	Uniprot id	Template	Identity (%)	RMSD	TM score	Ramachandran plot	Proposed function
1.	P44506	PLP-binding protein, 1W8G	61	0.223	0.6229	99.5 % (94.4 % core 5.1 % allow 0.0 % gener 0.5 % disall)	Alanine racemase
		Pyridoxal phosphate-binding protein, 3SY1	57	0.243	0.5130		
		Alanine racemase, 4A3Q (HHpred result)	N/A	3.997	0.2651		
2.	P44641	Lysine-2,3-aminomutase, 2A5H	34	0.241	0.3718	99 % (90.8 % core 8.2 % allow 0.0 % gener 1.0 % disall)	Lysine 2,3-aminomutase
3.	P46494 (Robetta server)	Rosetta de novo protocol (no template used)	N/A	N/A	N/A	100 % (86.7 % core 13.3 % allow 0.0 % gener 0.0 % disall)	DNA Topoisomerase,type IA,Zn finger
4.	P44827	pseudouridine synthase Rlu E, 2OLW	66	0.233	0.73306	98.1 % (87.3 % core 9.9 % allow 0.9 % gener 1.9 % disall)	Pseudouridine synthase Rlu E
		pseudouridine synthase Rlu E, 2OML	66	0.604	0.74206		
		Ribosomal small subunit pseudouridine synthase A, 1KSK	31	1.662	0.71557		
5.	Q57151	AP endonuclease, family 2, 3NGF	54	0.206	0.8329	99.6 % (92.4 % core 7.1 % allow 0.0 % gener 0.4 % disall)	hydroxypyruvate isomerase/D-tagatose 3-epimerase
		Putative Oxygenase, 1K77	60	0.751	0.9661		
		L-ribulose 3-epimerase, 3VYL	26	1.759	0.4682		
6.	P44094	Nucleoside-diphosphate-sugar epimerase, 2HRZ	41	0.194	0.1688	99.6 % (92.8 % core 6.1 % allow 0.7 % gener 0.4 % disall)	Nucleoside-diphosphate-sugar epimerase
7.	P45104 (ITASSER)	Ribosomal large subunit pseudouridine synthase F, 3DH3	29	0.657	0.66428	97.8 % (81.9 % core 12.4 % allow 3.5 % gener 2.2 % disall)	Ribosomal large subunit pseudouridine synthase F
8.	P71373	Nucleoside-diphosphate sugar epimerase (SulA family), 3OH8	33	0.655	0.84610	99.6 % (93.2 % core 5.7 % allow 0.8 % gener 0.4 % disall)	Nucleoside-diphosphate sugar epimerase
		Epimerase family protein SDR39U1, 4B4O	32	0.572	0.92240		
9.	P44160	Crystal Structure Analysis of HI1317, 1JOV	93	0.227	0.97926	99.6 % (88.8 % core 9.5 % allow 1.2 % gener 0.4 % disall)	Galactose mutarotase (Aldose 1-epimerase)
		putative mutarotase (YeaD), 2HTA	33	0.640	0.94691		
		Hexose-6-phosphate mutarotase, 2CIR	26	1.583	0.84898		
10.	Q57152 (ITASSER)	Solution NMR Structure of protein YqcC, 2HGK	47	0.876	0.97784	96.9 % (85.4 % core 10.4 % allow 1.0 % gener 3.1 % disall)	Beta-fructofuranosidase/invertase inhibitor
11.	P44268	Crystal structure of a DUF692 family protein, 3BWW	73	0.478	0.79170	98.9 % (86.5 % core 11.3 % allow 1.1 % gener 1.1 % disall)	Xylose isomerase-like
		L-ribulose 3-epimerase, 3VYL	50	3.912	0.73310		
12.	P52606	Crystal structure of Escherichia coli DiaA, 2YVA	35	0.364	0.98496	99.4 % (92.7 % core 5.6 % allow 1.1 % gener 0.6 % disall)	Sedoheptulose 7-phosphate isomerase
		Phosphoheptose isomerase, 1X92	32	0.341	0.98528		
		Phosphoheptose isomerase, 3BJZ	32	0.447	0.87983		
		Phosphoheptose isomerase 1, 1TK9	27	0.495	0.92181		
		Phosphoheptose Isomerase, 2I2 W	28	0.521	0.86321

The DALI server shows high structure similarity of P44506 with proteins with functionality of alanine racemase (Table S6). We observed a significant match with lysine-preferred racemases (Z score = 20.9), alanine racemase (Z score = 20.8), etc. The aligned residues are usually in the range of 221–628 with RMSD in the range of 0.3–3.1 Å^2^, and similarity usually ranges from 12 to 62 %. We also observed a close structural similarity to d-serine dehydratase. Furthermore, ProFunc (Table S6) server revealed eight motifs in the InterPro (Mulder et al. 2002) database with pyridoxal 5′-phosphate-dependent enzyme motif. An extensive sequence and structure analyses strongly suggest that HP P44506 is a PLP-dependent alanine racemase. Alanine racemase is a PLP-dependent enzyme which is important for bacterial cell wall biosynthesis in which it catalyzes the inter-conversion of alanine enantiomers (Noda et al. 2004).

### HP P44641

HP P44641 is localized in cytoplasm and not involved in non-classical secretory pathway and lacking any transmembrane helix (Table S2). The sequence-based function prediction suggests the presence of lysine 2, 3-aminomutase activity in the HP P44641 (Table S3 and S4). The MEME suite also suggests that P44641 may have lysine 2, 3-aminomutase activity. We discovered three sequence motifs, namely 120′-GCAVNC, 236′-IFAHAM, and 88′-GFSTDP (Table 2). This HP is a non-virulent protein (Table S3). The predicted interaction partners of HP P44641 are elongation factor P, lysyl-tRNA synthetase, diaminopimelate decarboxylase, opacity-associated protein, glycogen phosphorylase, biotin synthase, lysyl-tRNA synthetase, acetate CoA-transferase beta subunit, opacity-associated protein OapB and 23S rRNA 5-methyluridine methyltransferase.

The sequence of HP P44641 was also annotated in the Unirpot database to explore its possible function. We found that HP P44641 is annotated as an enzyme l-lysine 2,3-aminomutase which produces (R)-beta-lysine from (S)-alpha-lysine (l-lysine). This protein has several cofactor binding sites including [4Fe–4S] cluster and PLP-binding motif. Family and domain database search analysis indicates that HP P44641 belongs to the radical sam superfamily kama family containing a signature motif CxxxCxxC. The characteristic three-cysteine motif nucleates a [4Fe–4S] cluster, which binds SAM as a ligand to the unique Fe not ligated to a cysteine residue (Frey et al. 2008). The members of this family participate in more than 40 distinct biochemical transformations, and most of the members are not characterized biochemically so far. GO analysis suggest that this is a protein which involved in metabolic process, possesses isomerase like catalytic activity, and a metal-binding protein which specifically binds to the 4 iron and 4 sulfur.

**Fig. 2:**
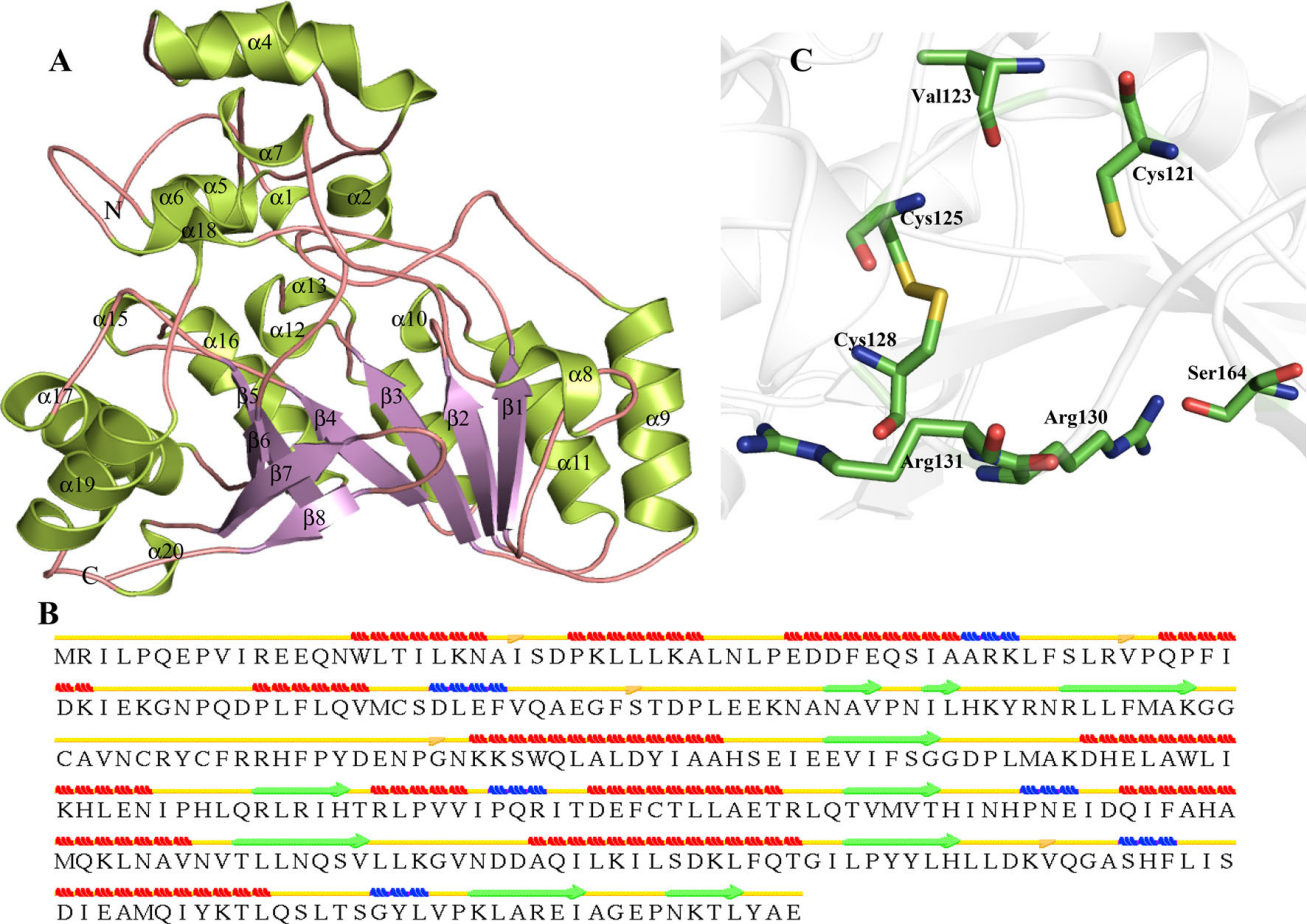
Representation of model structure of HP P44641. **a** Overall structure is represented in *cartoon*. **b** Secondary structure. **c** Predicted active site residues shown in *stick*

Structure of P44641 was predicted by MODELLER using lysine-2, 3-aminomutase (PDB ID–2A5H) as template. P44641 shows a sequence similarity of 34 % with 2A5H and TM score of 0.3718. The RMSD value after aligning target and template was found to be 0.241 Å^2^, indicating close structural similarity (Table 3). The predicted model of HP P44641 is comprised of (α/β)_8_ TIM barrel fold (Fig. 2a) containing eight β-strands in the barrel. The overall structure contains twelve α-helices, six 3_10_ helices and ten β-strands. P44641 also contains isolated β bridge at Ile24, Val55, Ser90 and Val291 (Fig. 2b). We observed three SAM-binding sites in this HP at Cys121, Cys125 and Cys128 (Table S5). We predicted that the active site residues of P44641 are Cys121, Val123, Cys125, Cys128, Arg130, Arg131 and Ser164 (Fig. 2c).

Furthermore, we observed a significant resemblance with ribosomal RNA large subunit methyltransferase N (Z score = 15.8, RMSD = 2.6 Å^2^), pyruvate formate-lyase 1-activating enzyme (Z score = 15.0, RMSD = 2.7 Å^2^), etc. Similarly, ProFunc shows that the predicted structure of P44641 contains nine characteristic motifs with function of lysine-2, 3-aminomutase and four significant ligand-binding templates. Our extensive analysis shows that P44641 contains lysine-2, 3-aminomutase activity. Since lysine is used as a source of energy in bacteria (Lepore et al. 2005). Hence, the lysine-2, 3-aminomutase, a radical SAM-dependent enzyme, performs the inter-conversion of L-α-lysine and L-β-lysine, the elementary step in lysine degradation (Lepore et al. 2005) in the bacteria.

### HP P46494

HP P46494 is predicted to be localized in cytoplasm and periplasm as suggested by PSLpred and CELLO, respectively (Table S2). This protein is secretory in nature but lacks signal peptide and transmembrane helix. The function analysis reveals that the HP P46494 comprises DNA topoisomerase activity (Table S3 and S4). The INTERPROSCAN and MOTIF tools identified domain with a function of DNA topoisomerase (type IA, Zn finger). This prediction is further confirmed by MEME suite, which identified three signature sequences in P46494, namely 76′-FGMFIGCSHYPECDFVV, 1′-MNQSLFHH, 115′-RRGRQGKIFY a signature sequence for DNA topoisomerase I, a zinc metalloprotein with three repetitive zinc-binding domains (Tse-Dinh and Beran-Steed 1988). This protein is non-virulent and involved in cellular processes (Table S3). STRING database suggests several interaction partners such as DNA topoisomerase III, shikimate 5-dehydrogenase, ABC transporter ATP-binding protein, DNA-3-methyladenine glycosylase, DNA processing chain A, recombination regulator RecX, peptide deformylase, methionyl-tRNA formyltransferase and recombinase A. Gene ontology analysis suggests that HP P46494 is involved in the DNA binding and causes a topological change in the DNA; hence, it has type 1 DNA topoisomerase-like activity.

**Fig. 3:**
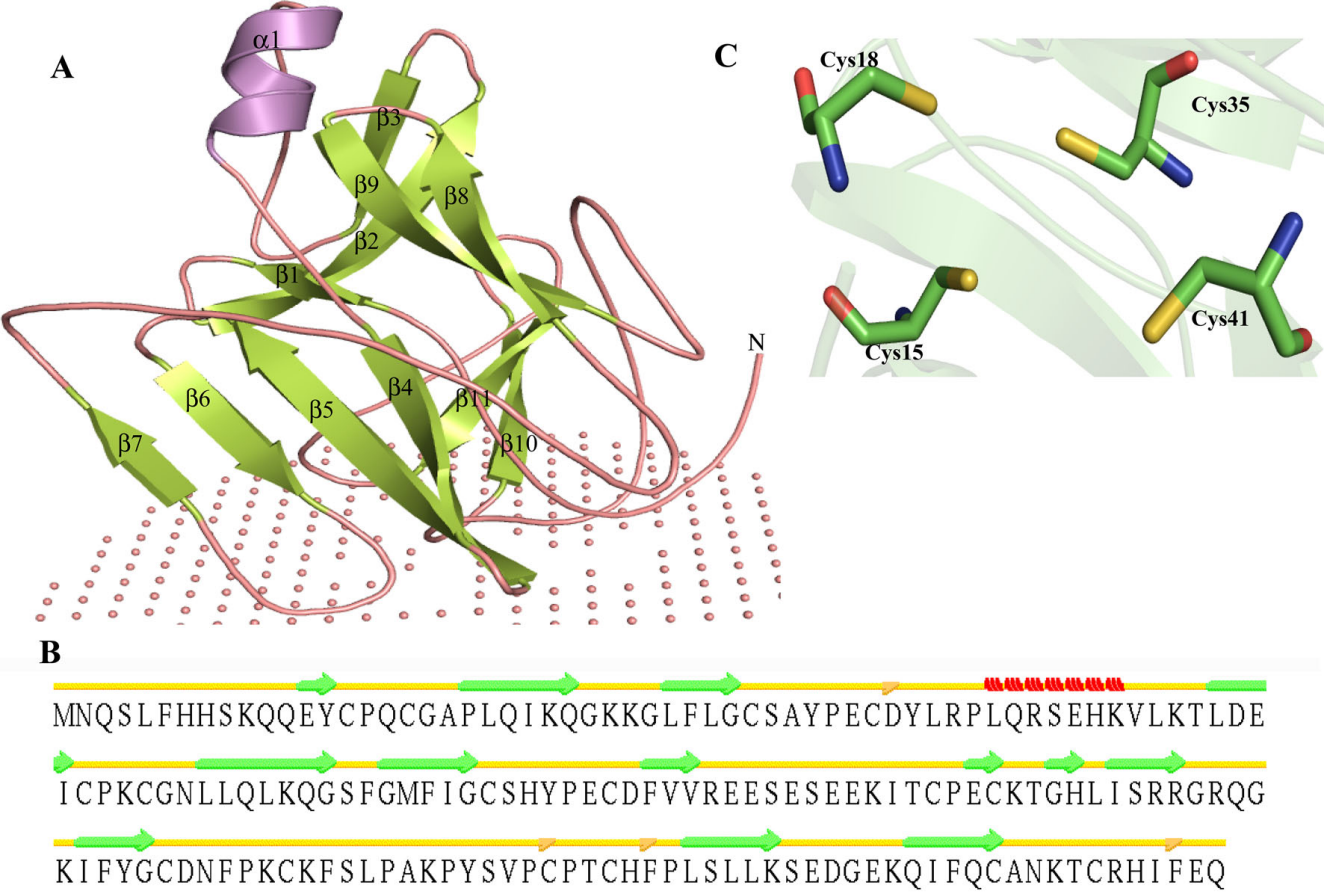
Representation of model structure of HP P46494. **a** *Cartoon model* showing overall structure with non-bonded sphere describing the membrane. **b** Secondary structure of P46494. **c** A detailed description of P46494 active site

Due to the unavailability of any reliable template in the PDB, we were unable to predict the structure of HP P46494 using homology modeling. Here, we used Robetta server for the prediction of structure of P46494 using the Rosetta de novo protocol. The predicted model shows most of the residue in the allowed region of Ramachandran plot (Table 3). Overall structure is similar to domain II of DNA topoisomerase type I (Champoux 2001) (Fig. 3a). The secondary structure prediction shows that HP P46494 consists of 13 β-strands and single α-helix (Fig. 3b) of seven residues (Leu47, Gln48, Arg49, Ser50, Glu51, His52 and Lys53). Isolated β-bridges are present at Asp42, Cys145, Phe150 and Phe176 (Figure S4). We observed zinc-binding sites at Cys15, Cys18, Cys35, Cys41, Cys104, Cys107, Cys145 and Cys148 (Table S5). Extensive analysis of P46494 predicted that active site may consist of Cys15, Cys18, Cys35 and Cys41 (Fig. 3c).

We also identified Pro151 is a membrane-embedded residue according to PPM server which calculates rotational and translational positions in a protein structure. The twisting in topoisomerase is essential for its biological activity and Pro151 is one of the essential residues for such conformational changes in this enzyme during catalysis. The structure similarity using DALI server shows a model which is similar to 2GAI only (Z score = 0.4, RMSD = 6.0 Å^2^) (Table S6). ProFunc has identified three motifs as zf-C4_Topoisom, etc. Further, six ligand-binding templates are also recognized in reference to P46494. These analyses suggest that P46494 is a DNA topoisomerase IA (Zn finger)-like protein. DNA topoisomerase type IA has an exclusive mechanism of strand passage over an enzyme-bridged, ssDNA gate, consequently allowing them to carry out varied reactions in processing structures crucial for replication, recombination and repair (Lee et al. 2013).

### HP P44827

HP P44827 is localized in the cytoplasm, lacks any transmembrane helix and is not involved in any secretory pathway (Table S2). HP P44827 contains ribosomal large subunit pseudouridine synthase E activity as suggested by sequence analysis (Table S3 and S4). The MEME suite also predicted a similar function for HP P44827 along with the three annotated motifs 84′-VYAAGRLDRDSEGLLILTNNGELQHRLADPKFKTEKTYWVQVEGI, 51′-TKVVLFNK PFDVLTQFTDEQGRATLKD, and 178′-WLEIKISEGRNRQVRRMTAHIGFP (Table 2). Uniprot has also annotated this HP as ribosomal large subunit pseudouridine synthase E (rluE) which is responsible for synthesis of pseudouridine from uracil-2457 in 23S ribosomal RNA. Such enzymes catalyze the isomerization of specific uridines in an RNA molecule to pseudouridines (5-ribosyluracil, psi). The domain surface is populated by conserved, charged residues that define a likely RNA-binding site. Further, P44827 is involved in metabolism and a non-virulent protein (Table S3). The STRING database suggests that HP P44827 interacts with lipoprotein E, β-hexosaminidase, 23S rRNA pseudouridylate synthase C, adenylosuccinate lyase, transport protein and tRNA-specific 2-thiouridylase MnmA.

**Fig. 4:**
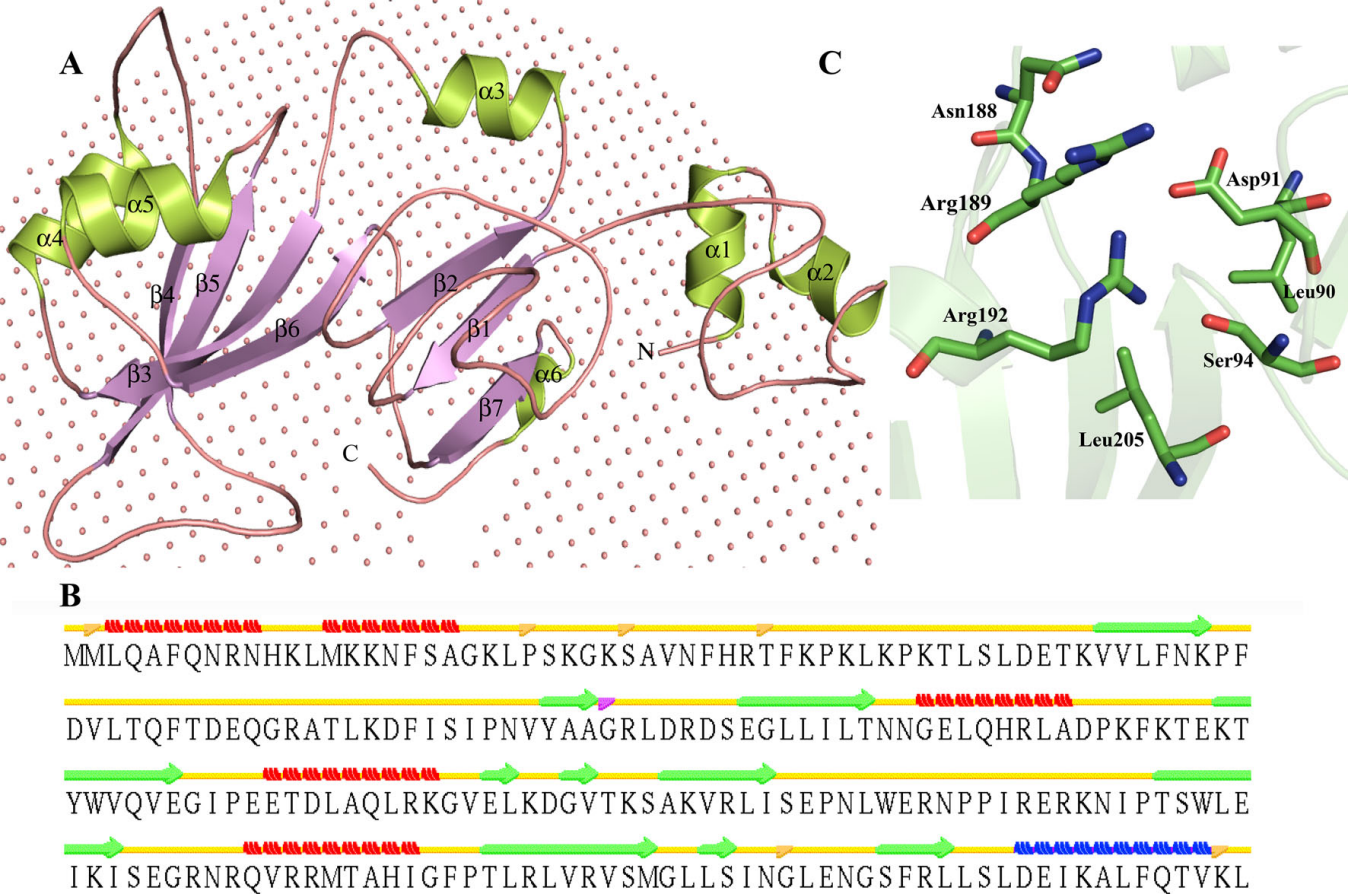
Representation of model structure of HP P44827. **a** *Cartoon diagram* showing mixed alpha/beta fold. Collection of non-bonded spheres represents membrane. **b** Secondary structure evaluation using predicted three-dimensional structure. **c** Stick representation of P44827 active site, with Asp91 residue is proposed to be involve in nucleic acid binding

Three-dimensional structure of P44827 was predicted by MODELLER (Fig. 4a) using pseudouridine synthase Rlu E (PDB ID—2OLW), pseudouridine synthase Rlu E (PDB ID—2OML) and ribosomal small subunit pseudouridine synthase A (PDB ID—1KSK) as templates with sequence identity of 66, 66 and 31 %, respectively (Table 3). The refined model shows RMSD of 0.233, 0.604 and 1.662 with their templates 2OLW, 2OML and 1KSK, respectively, indicating closer structural and functional similarity. The calculated TM scores between templates and target were found to be 0.73306, 0.74206 and 0.71557, respectively, which further support the functional similarity. Overall structure of HP P44827 adopts an α/β-fold attribute bifurcated, typically antiparallel β-sheet, present in all Ψ synthases. It also contains four conserved helices, i.e., three α-helices and one 3_10_-helix that group next to the β-sheets (Fig. 4b) with an additional α-helix. We found only three central strands of β-sheet, namely, β2, β3 and β6, instead of four strands that form the floor of the cleft (Fig. 4a). These strands are highly conserved in Ψ synthases, and cleft certainly contains an active site of pseudouridine synthase enzyme. An isolated beta bridges was observed at Met2, Pro24, Ser29, Thr36 and Gly217 (Fig. 4c). The residues Ile231, Leu234, Gln236, Thr237 and Leu240 are found to be embedded in membrane. Active site analysis suggested that Asp91 is essential for function of this enzyme. Further analysis revealed the active site of HP P44827 contains Leu90, Asp91, Ser94, Asn188, Arg189, Arg192 and Leu205 (Fig. 4c).

Structure similarity searches clearly indicates that HP P44827 has a close structure similarity to the small subunit of pseudouridine synthase (Z score = 23.7, RMSD = 3.3 Å^2^), and hence this protein may possess pseudouridine synthase-like activity. We found a similar structural pattern with six pseudouridine synthase on ProFunc analysis. These observations suggest that HP P44827 may be a pseudouridine synthase E. There are five characterized subfamilies of Ψ synthases in prokaryotes on the basis of sequence conservation (Gustafsson et al. 1996). The pseudouridine synthase RluE is classified as a member of RsuA family (Del Campo et al. 2001) and modifies the single site Ψ2457 on a stem of 23S RNA.

### HP Q57151

The sequence analysis showed that the HP Q57151 is localized in cytoplasm and is not involved in secretory mechanisms (Table S2). Sequence-based function analysis clearly indicates that HP Q57151 is a hydroxypyruvate isomerase and a non-virulent protein (Table S3 and S4). We identified three motif repeats in HP Q57151 as 99′-CPNVHIM, 71′-WGGSAI, 78′-DYFHAQ (Table 2). The predicted functional partners for Q57151 are 3-hydroxyisobutyrate dehydrogenase, putative aldolase, glycerate dehydrogenase, glycerol-3-phosphate regulon repressor, gluconate permease, D-xylose transporter subunit XylF and cAMP-regulatory protein indicating its importance for the survival of the organism.

Uniprot annotation suggests that HP Q57151 is a putative hydroxypyruvate isomerase which catalyzes the reversible isomerization between hydroxypyruvate and 2-hydroxy-3-oxopropanoate. Domain annotation suggests that HP Q57151 contains a structural motif with a beta/alpha TIM barrel which is found in several proteins families including xylose isomerase. Family analysis suggests that HP Q57151 belongs to the hydroxypyruvate isomerase Hyi and possesses hydroxypyruvate isomerase activity.

**Fig. 5:**
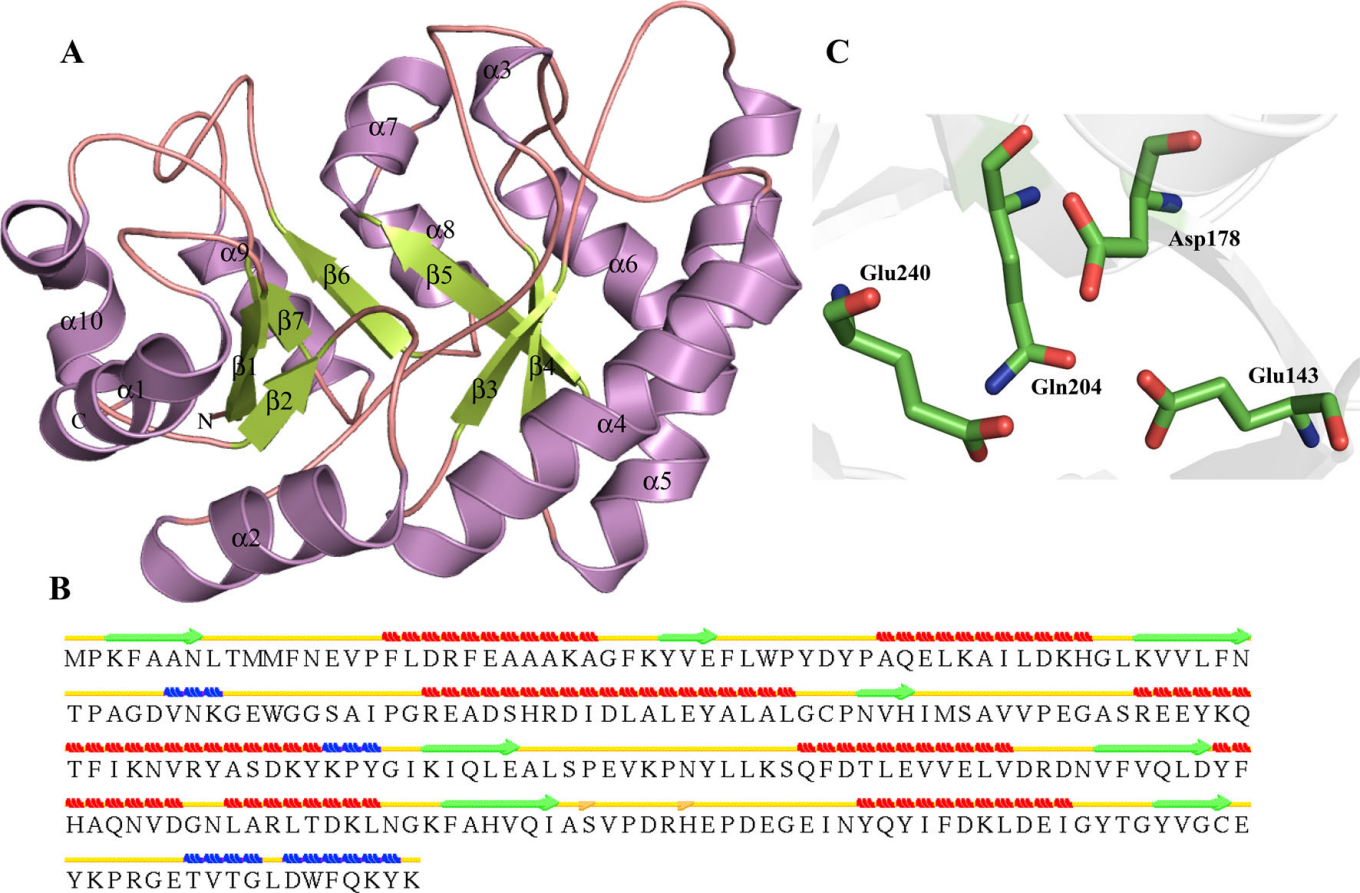
Representation of model structure of HP Q57151. **a** *Cartoon model* showing overall topology described by predicted structure. **b** Description of secondary structure prediction in the HP. **c** A detailed description of active site

The AP endonuclease family 2 protein (PDB ID—3NGF), putative oxygenase (PDB ID—1K77), L-ribulose 3-epimerase (PDB ID—3VYL) are used as templates by MODELLER for the prediction of a model for Q57151. The model is showing 99.5 % residues in the allowed region of Ramachandran plot (Table 3). The overall structure is comprised of a TIM barrel fold (Table 3; Fig. 5a) (Gerlt and Raushel 2003; Wierenga 2001). Overall structure consists of eight αβ-fold unit, with eight parallel β-strands located in the interior and eight α-helices on the exterior of the barrel. Instead of (α/β) eightfold we observed seven β-sheets in TIM barrel. Furthermore, two isolated β-bridges are observed at Ser207 and His212 (Fig. 5b). The active sites are located at C terminal end of β strand in αβ loops of TIM barrel (Fig. 5a). The manganese-binding sites are located at the C-terminal ends of β-strands. We predicted Glu143, Asp178, Gln204 and Glu240 as important residues for binding (Fig. 5c). These predictions are supported by structure-based active site prediction servers (Table S5).

The predicted structure of HP Q57151 is quite similar to those of D-tagatose 3-epimerase (Z score = 26.1, RMSD = 2.3 Å^2^), L-ribulose 3-epimerase (Z score = 26.1, RMSD = 2.4 Å^2^), etc., indicating that this HP may act as an epimerase. Moreover, structure-based function prediction using ProFunc shows this protein may acts as hydroxypyruvate isomerase, xylose isomerase-like, etc. All these finding suggest that the HP Q57151 is actually hydroxypyruvate isomerase which catalyzes a reversible conversion of hydroxypyruvate from tartronate semialdehyde (de Windt and van der Drift 1980).

### HP P44094

HP P44094 is a cytoplasmic, non-virulent and non-secretory protein (Table S2). We observed that HP P44094 contains a nucleoside-diphosphate-sugar epimerase domain (Table S3 and S4). MEME suite analysis suggests the presence of three significant motifs in the sequence of Q57151 namely 149′-MCELLINDYSRKGFVDGIVVRLPTICIRPGKPNKAASSFVSSIMREPLHG, 55′-CPVSEE and 291′-QALALGFKV (Table 2). STRING analysis suggests that gluconate permease, putative aldolase, 3-hydroxyisobutyrate dehydrogenase and glycerol-3-phosphate regulon repressor are the functional network partner of HP P44094. Sequence similarities search suggest that HP P44094 belongs to the NAD(P)-dependent epimerase/dehydratase family. However, a detail annotation of this HP is not available at the Uniprot.

**Fig. 6:**
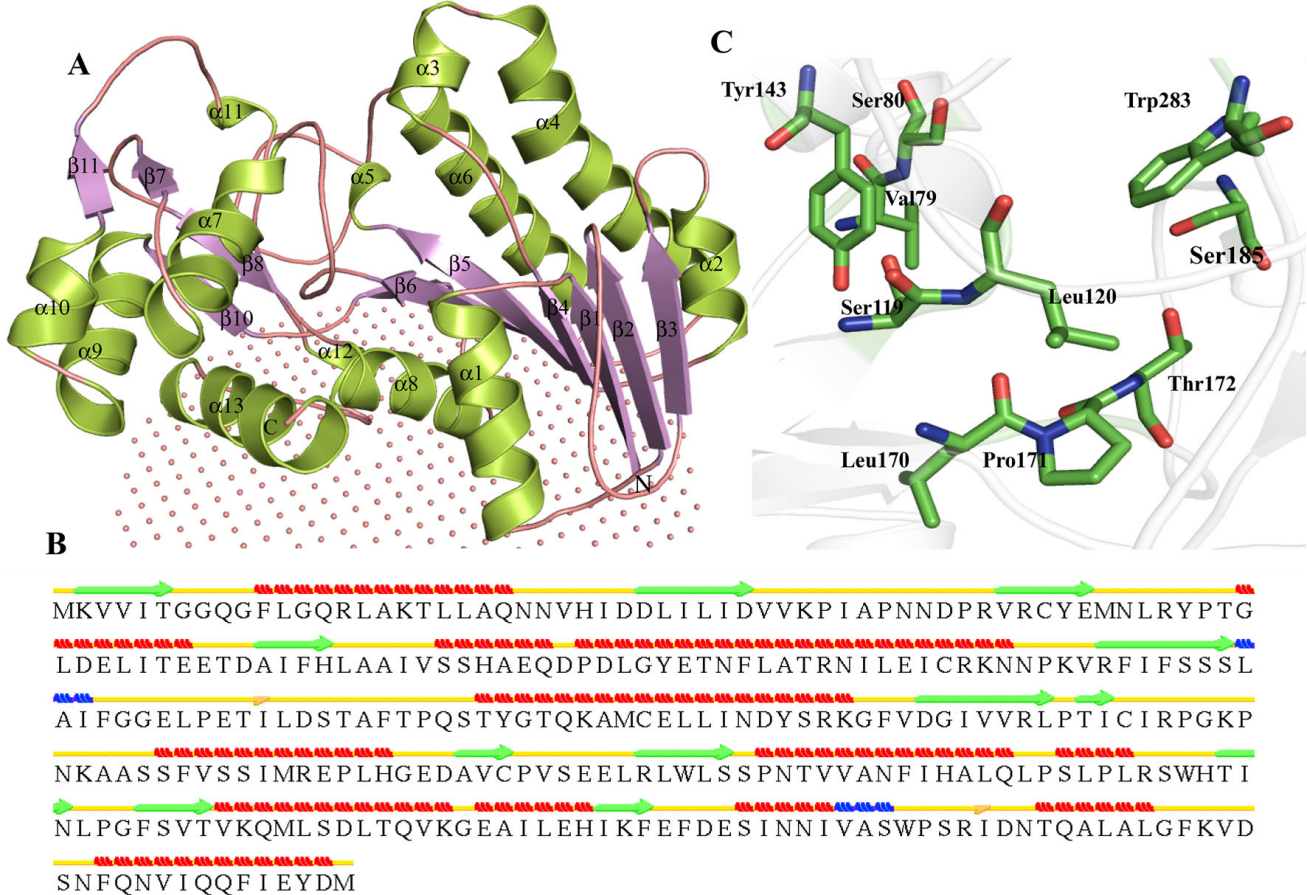
Representation of model structure of HP P44094. **a** Overall structure of P44094 shown in *cartoon model* with membrane is represented as non-bonded spheres. **b** Secondary structure of HP P44094. **c** Representation of the active site residues of P44094 in *stick model*

Structure of HP P44094 was modeled using nucleoside-diphosphate-sugar epimerase (PDB ID—2HRZ) as templates. The target and template showed a sequence identity of 41 % and RMSD of 0.194 Å^2^ indicating a close structural similarity (Table 3). The overall structure of HP P44094 contains 12 β-strands, 13 α-helices and two 3_10_ helices (Fig. 6a). There are two isolated β-bridges at Ile131 and Ile287 (Fig. 6b). We observed an N-terminal NAD-binding Rossmann-fold domain which spans over β1–β7 and α1–α8. Active site prediction analysis shows that Tyr143 is responsible for the activity of HP P44094 (Table S5). The active site may contain Val79, Ser80, Ser119, Leu120, Tyr143, Leu170, Pro171, Thr172, Ser185 and Trp283 (Fig. 6c). The Leu232 and Pro233 are found to be membrane-embedded residues. The structure similarity analysis shows high similarity with NDP-sugar epimerases with z score in the range 32.8–33.4 and RMSD of 2.6 Å^2^. Further analysis shows the presence of NAD (P)-binding Rossmann-fold domains and NAD-dependent epimerase/dehydratase activity. On the basis of sequence and structure analyses, we successfully annotated the function of P44094 as nucleoside-diphosphate-sugar epimerase (UDP-glucose 4-epimerase). UDP-glucose 4-epimerase catalyzes the reversible inter-conversion of UDP-glucose and UDP-galactose which results in the formation of glucose- and galactose-containing exopolysaccharides (Dormann and Benning 1998).

### HP P45104

HP P45104 is localized in cytoplasm and lacks signal peptide (Table S2). It contains the domain with activity of ribosomal large subunit pseudouridine synthase (Table S3 and S4). The MEME suite analysis shows the presence of three significant motifs namely 176′-WIAVGRLDINTSGLLLFTTDGELANRLMHPSREVEREYSVRV FGQ, 140′-CRVLMYYKPEGELCTRSDPEGRATVFD and 256′-WYDVTLMEGRNREVRRLWESQGIQ, indicating a functional resemblance with ribosomal large subunit pseudouridine synthase B (Table 2). This protein is also annotated as rluB in the Uniprot database and belongs to the pseudouridine synthase RsuA family. Interaction networking partners of HP P45104 are 23S rRNA pseudouridine synthase D, transcriptional regulator CysB, 23S rRNA pseudouridylate synthase C, tRNA pseudouridine synthase B, GTP-binding protein EngA, 30S ribosomal protein S1 and cytidylate kinase. This also confirms its predicted function.

**Fig. 7:**
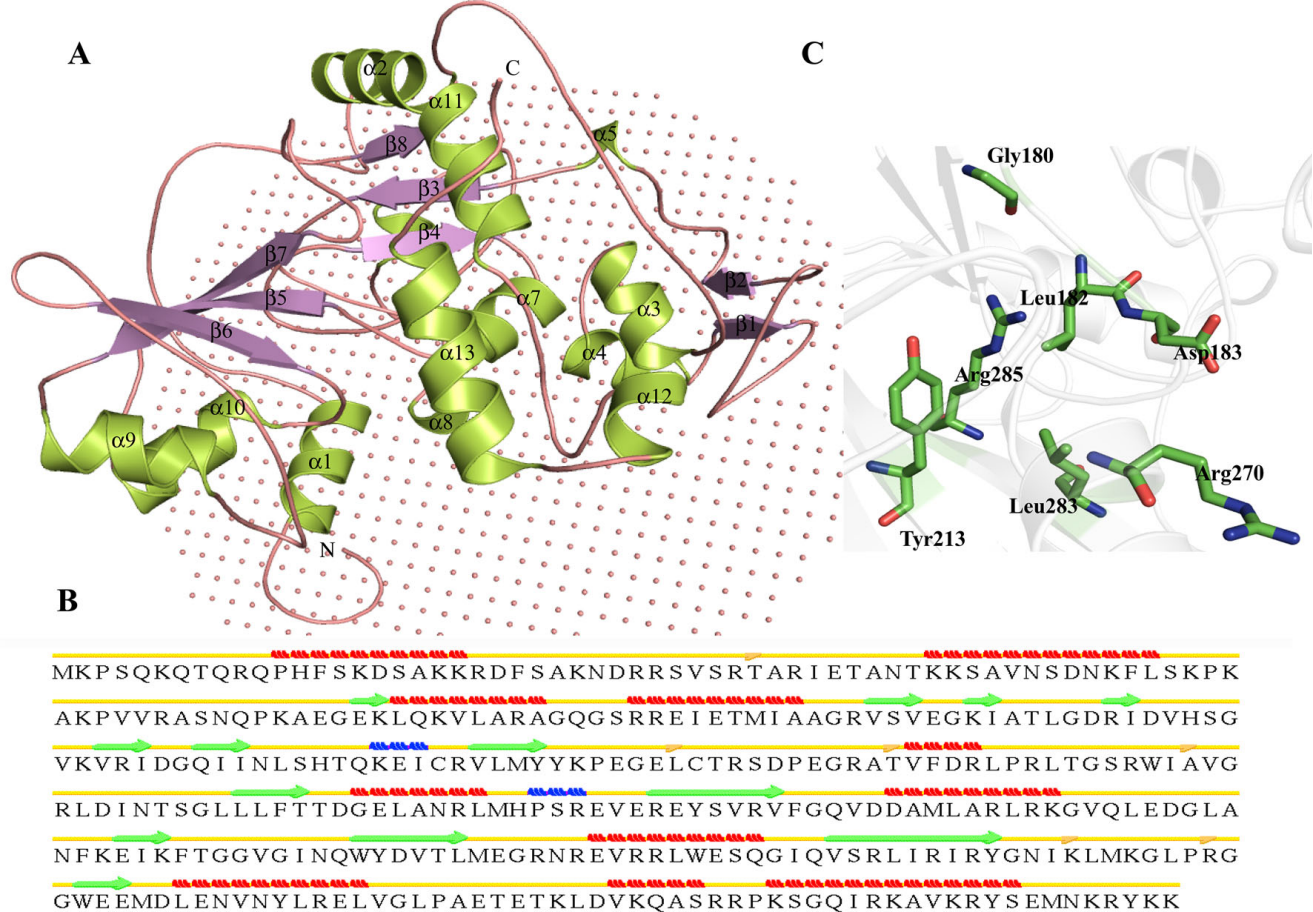
Representation of model structure of HP P45104. **a** Three-dimensional structure represented in *cartoon model* with membrane represented as *non-bonded spheres*. **b** Representation of secondary structure predicted using STRIDE. **c** Representation of the active site residues of HP P45104 in *stick model*

Here, we used ITASSER server for the prediction of structure of HP P45104. We found 97.8 % residues of P45104 are present in the allowed region of Ramachandran plot. The TM score was found to be 0.66428, indicating the predicted structure contains the similar fold present in ribosomal large subunit pseudouridine synthase F (PDB ID- 3DH3). The structure analysis shows 11 α-helices, 13 β-strands and two 3_10_-helix in the structure of HP P45104 (Fig. 7a). The presence of isolated β-bridges is found at Thr36, Leu152, Thr163, Ala178, Lys292 and Arg299 (Fig. 7b). The structure contains an N-terminal S4 domain or α-L RNA-binding motif (77–171) which connects through a linker to catalytic domain (142–309). The active site structure of P45104 adopts mixed α/β fold, which is common in all Ψ synthases. There are eight-stranded anti-parallel bifurcated β-sheet flanked by loops. The cleft of the active site is located in the center of the β-sheet in P45104. The active site contain conserved residue Asp183 which is essential for the activity of enzyme (Table S5). We predicted active site residues Gly180, Leu182, Asp183, Tyr213, Arg270, Leu283 and Arg285 in the structure of HP P45104 (Fig. 7c). The P45104 shows Ala99 to be a membrane-embedded residue.

HP P45104 shows a close resemblance with the ribosomal large subunit pseudouridine synthase B and ribosomal large subunit pseudouridine synthase F. These findings are clearly indicating that HP P45104 may functions as a large subunit pseudouridine synthase B. This enzyme catalyzes the conversion of U2605 to pseudouridine (Ψ) in a stem-loop of 23S rRNA (Czudnochowski et al. 2013), while close homolog RluF isomerizes the adjacent nucleotide in the stem, i.e., U2604 (Czudnochowski et al. 2013).

### HP P71373

HP P71373 was predicted as a virulent protein localized in the cytoplasm (Table S2). This protein is also a non-secretory protein and lacks transmembrane helix. The function prediction shows that HP P71373 may be an epimerase amidophosphoribosyltransferase (Table S3 and S4). Motif analysis also suggests the presence of epimerase activity in the HP P71373 (Table 2). HP P71373 was also annotated as epimerase family protein HI_1208 in the uniprot database and belongs to the NAD(P)-dependent epimerase/dehydratase family. The STRING predicts arginine repressor, malate dehydrogenase, ferrochelatase, lipoyltransferase, 2-oxoglutarate dehydrogenase E2 component dihydrolipoamide succinyltransferase and dihydrolipoamide acetyltransferase as functional networking partners.

**Fig. 8:**
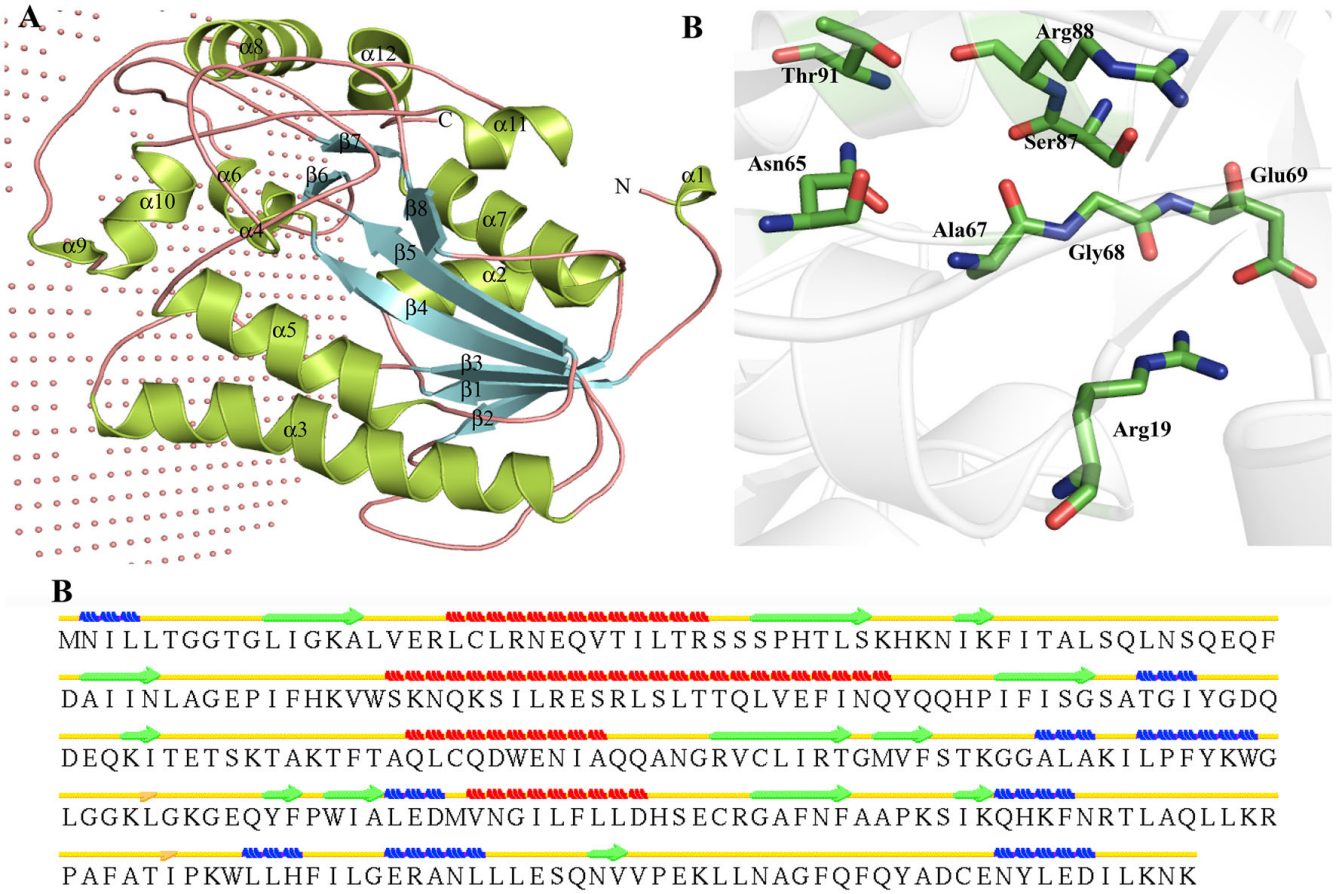
Representation of model structure of HP P71373. **a** Cartoon model representation of overall structure in which membrane is represented by *non-bonded atoms*. **b** Predicted secondary structure. **c** Representation of the active site residues of HP P71373 in *stick model*

The BLASTp suggests that HP P71373 is homologous to nucleoside-diphosphate-sugar epimerase (PDB ID-3OH8) and epimerase family protein SDR39U1 (PDB ID-4B4O). Hence, we used MODELLER for the structure prediction (Fig. 8a). The TM score of templates is >0.8 and low RMSD value indicates their high fold similarities with the model of HP P71373. Secondary structure analysis revealed four α-helices, 13 β-strands and nine 3_10_-helix (Fig. 8b). HP P71373 is comprised of NAD (P)-binding Rossmann-fold domains (1-293). The active pocket of HP P71373 contains Arg19, Asn65, Ala67, Gly68, Glu69, Ser87, Arg88 and Thr91 (Fig. 8c; Table S5). The membrane rooted residues are Val75, Pro175, Trp179, Gly180, Leu181, Pro241, Phe243, Ala244, Thr245, Ile246, Pro247, Trp249, Leu250, Leu251, Phe253 and Ile254 as suggested by the PPM server. The P71373 is revealed to be a UDP-glucose 4-epimerase after comparing its structure with proteins present in the PDB. The structure-based function analyses clearly indicates that HP P71373 may be a nucleoside-diphosphate-sugar epimerase (UDP-glucose 4-epimerase).

### HP P44160

HP P44160 is a secretory protein present in the cytoplasm (Table S2). There is no transmembrane helix present in the sequence of P44160. The motif and domain analysis suggests that the HP P44160 is an aldose 1-epimerase enzyme which is important for metabolic pathways like glycolysis and gluconeogenesis (Chittori et al. 2007) (Table S3 and S4). Uniprot annotation has also indicated that HP P44160 is a putative glucose-6-phosphate 1-epimerase which converts α-d-glucose 6-phosphate to β-d-glucose 6-phosphate. Furthermore, GO analysis indicated that this protein is involved in the carbohydrate metabolic process. Interestingly, sequence similarity search also suggest that this HP belongs to the glucose-6-phosphate 1-epimerase family. The HP P44160 is a virulent protein involved in cellular process. It contains three motifs predicted by MEME suite, namely 86′-QPAHGT, 75′-PICYPW and 29′-CGWNTKNFPC (Table 2). The predicted partners for P44160 are glucose-6-phosphate isomerase, glucose-specific PTS system component, keto-hydroxyglutarate-aldolase/keto-deoxy-phosphogluconate aldolase, transaldolase B, deoxyribose-phosphate aldolase, transketolase, fructose-bisphosphate aldolase, aldose 1-epimerase and UDP-glucose 4-epimerase, indicating the role HP P44160 in carbohydrate metabolism.

**Fig. 9:**
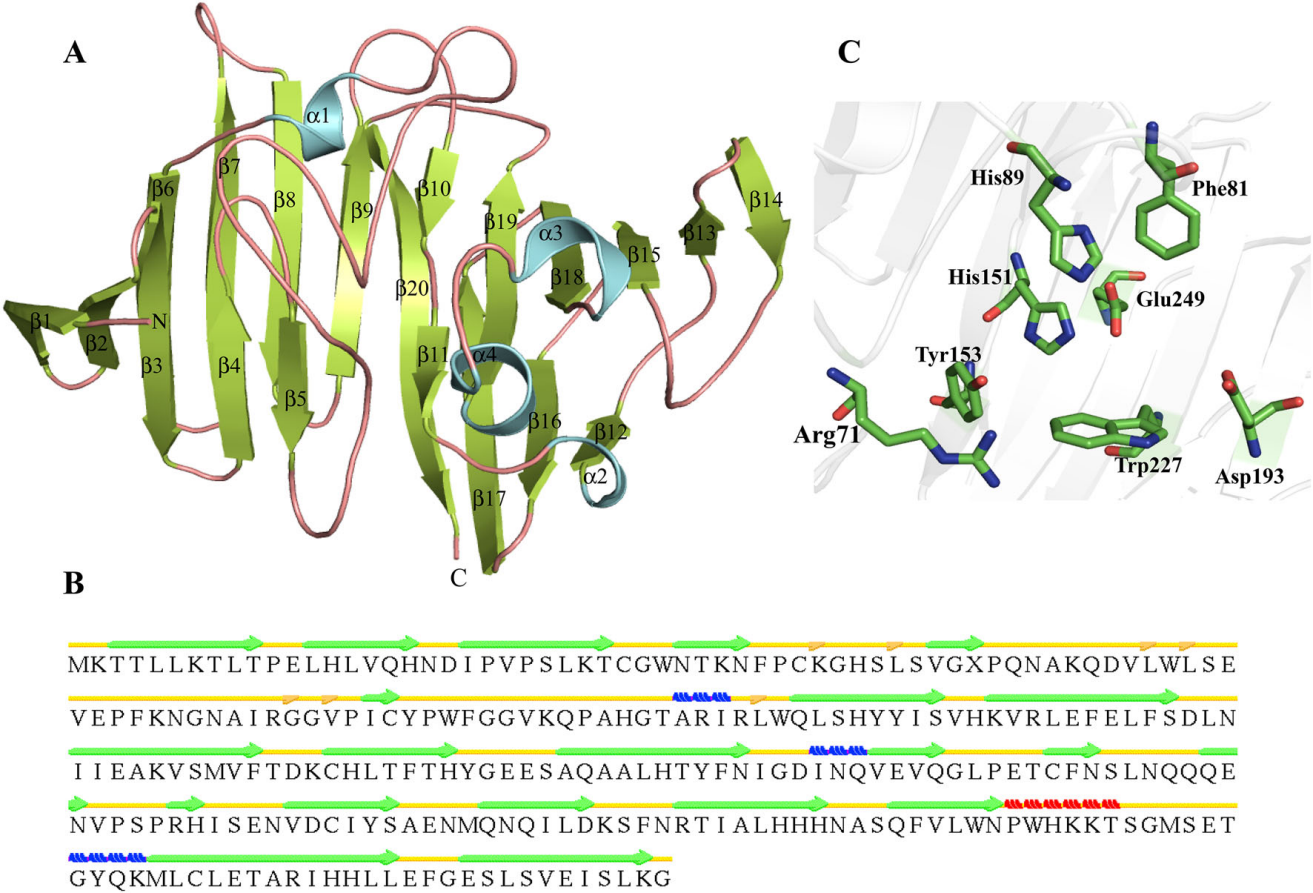
Representation of model structure of HP P44160. **a** Showing characteristic β-sandwich topology. **b** Detailed description of secondary structure using STRIDE. **c** The active site pocket is illustrated in *stick* representation

We used MODELLER for structure prediction of HP P44160 using putative mutarotase (PDB ID-2HTA) and hexose-6-phosphate mutarotase (PDB ID-2CIR) as templates. The predicted model shows 99.6 % residues in the allowed region and very high fold similarity with the templates. The structure of HP P44160 adopts a β-sandwich fold made up of 21 β-strands, one α-helix and three 3_10_-helices (Fig. 9a, b). All 20 β-strands are arranged in three anti-parallel β-sheets in P44160. The three β-sheets are organized in two layers. The first layer consists of two sheets S1 (β1–β5) and S3 (β13–β20), while the other layer contains S2 (β6–β12). The α3 and α4 are present on the same side connecting β18 to β19, while α1 connects β5 to β6 and α2 connects β11 to β12 (Fig. 9a). The active site is a β-D-galactose binding pocket that contains Arg71, Phe81, His89, His151, Tyr153, Asp193, Trp227 and Glu249 (Fig. 9c). The DALI search shows that the structure of HP P44160 is highly similar to those of epimerases like hexose-6-phosphate mutarotase (Z score = 31.6, RMSD = 2.0 Å^2^), glucose-6-phosphate 1-epimerase (Z score = 31.5, RMSD = 2.0 Å^2^) etc. Similarly, ProFunc also shows that HP may contain epimerase activity. The aldose 1-epimerase are the enzymes that catalyze the anomeric inter-conversion of aldose sugars like d-glucose, etc., into their α and β forms (Graille et al. 2006).

### HP O86237

The HP O86237 is a cytoplasmic protein showing tautomerase/MIF activity (Table S2 and Table 3). GO annotation has also indicated that HP O86237 is involved in the cellular aromatic compound metabolic process and possesses isomerase activity. Interestingly, family and domain database search has also indicated that this HP belongs to the 4-oxalocrotonate_tautomerase family. These predictions were further confirmed by understanding the interaction network of O86237 using STRING database which shows HP O86237 interacts with anthranilate phosphoribosyl transferase, bifunctional indole-3-glycerol phosphate synthase/phosphoribosylanthranilate isomerase, anthranilate synthase component II and anthranilate synthase component I.

**Fig. 10:**
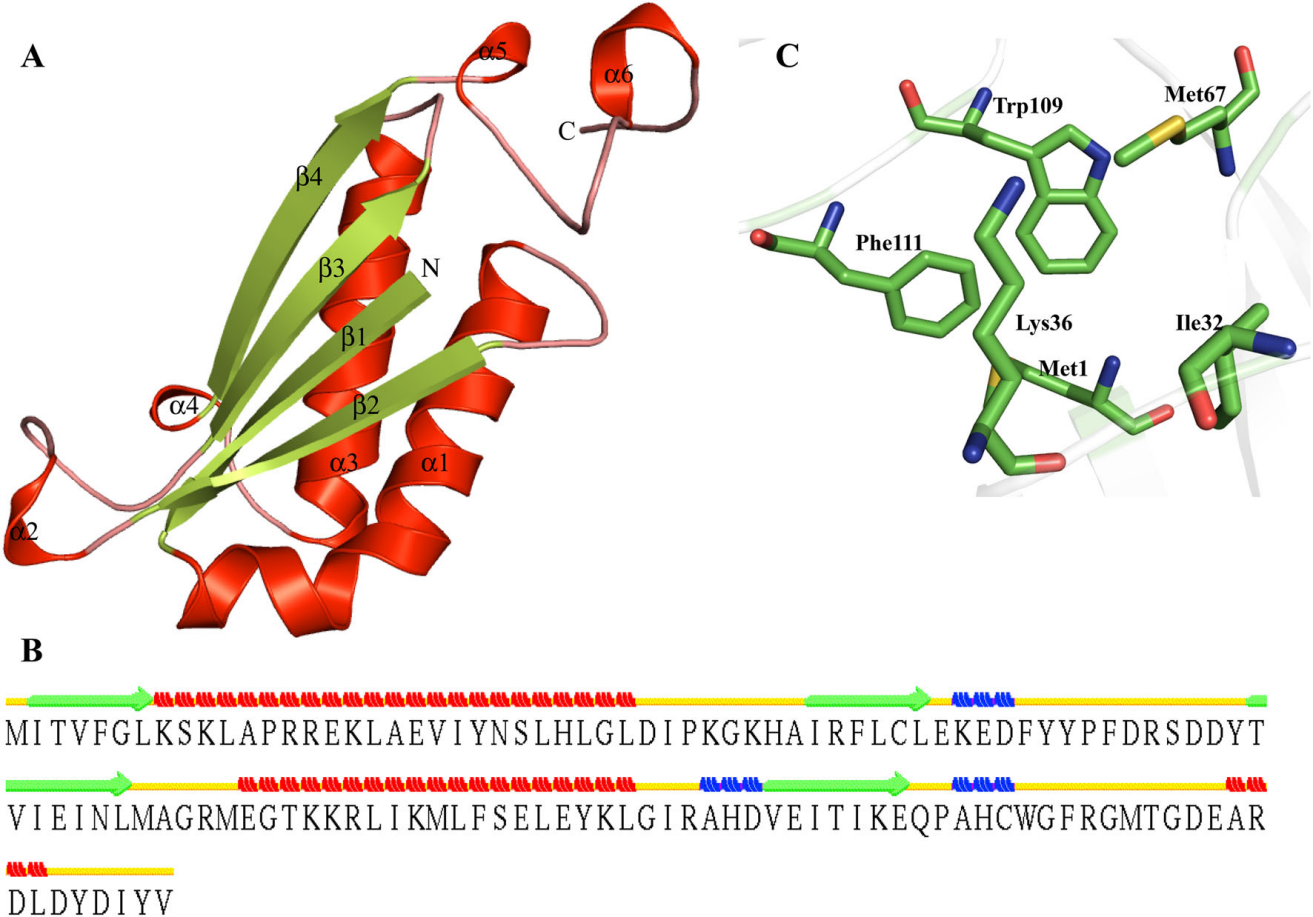
Representation of model structure of HP O86237. **a** Overall structure is represented in *cartoon form*. **b** Secondary structure of HP O86237. **c** Detailed description of active site of O86237

The crystal structure of HP O86237 has been determined (PDB id: 1MWW) comprised of a tautomerase MIF fold. HP O86237 shows a close similarity to putative 4-oxalocrotonate tautomerase (PDB ID 4LKB), malonate semialdehyde decarboxylase (PDB ID—3MLC), malonate semialdehyde decarboxylase (PDB ID—4LHP) and macrophage migration inhibitory factor (PDB ID—4DH4). The O86237 shows the presence of three α-helices, four β-strands and three 3_10_-helices (Fig. 10a). We observed a β-α-β fold in the predicted model of HP O86237 (Fig. 10b). This fold is a characteristics of tautomerase superfamily which includes members like macrophage migration inhibitory factor (MIF) and D-dopachrome tautomerase. (Almrud et al. 2002). The active site of HP O86237 contains Met1, Ile32, Lys36, Met67, Trp109 and Phe111 (Fig. 10c). DALI server further indicates that HP O86237 is structurally similar to the malonate semialdehyde decarboxylase (Z score = 18.0, RMSD = 1.6 Å^2^), putative tautomerase (Z score = 15.3, RMSD = 1.9 Å^2^), etc. Moreover, ProFunc analysis suggests that HP O86237 may have tautomerase/MIF function. These findings help us to propose the function of HP O86237 as a tautomerase/MIF, a key regulatory cytokine of innate and adaptive immune responses (Donn and Ray 2004).

### HP Q57152

The PSLpred server shows that HP Q57152 is localized in periplasm, while CELLO suggests cytoplasmic localization (Table S2). This is a virulent protein involved in cellular processes with tRNA pseudouridine synthase C activity (Table S3 and S4). Uniprot annotation has also indicated that HP Q57152 is similar to the N-terminal of *E. carotovora* exoenzyme regulation regulon ORF1 and the C-terminal part is colinear with YqcB. YqcC-like structural domain found in the N-terminal of some tRNA pseudouridine synthase C proteins, as well as other uncharacterised proteins.

Results were validated by STRING which shows interaction network contains putative sulfate transport protein CysZ, penicillin-binding protein 1B, N-acetylmuramic acid-6-phosphate etherase, opacity protein, anhydro-N-acetylmuramic acid kinase, ATP-dependent helicase along with Q57152. These predictions are further validated by MEME suite which identified three sequence-based motifs namely, 51′-WVFIPRM, 72′-AISPYI and 38′-FSIDTM.

**Fig. 11:**
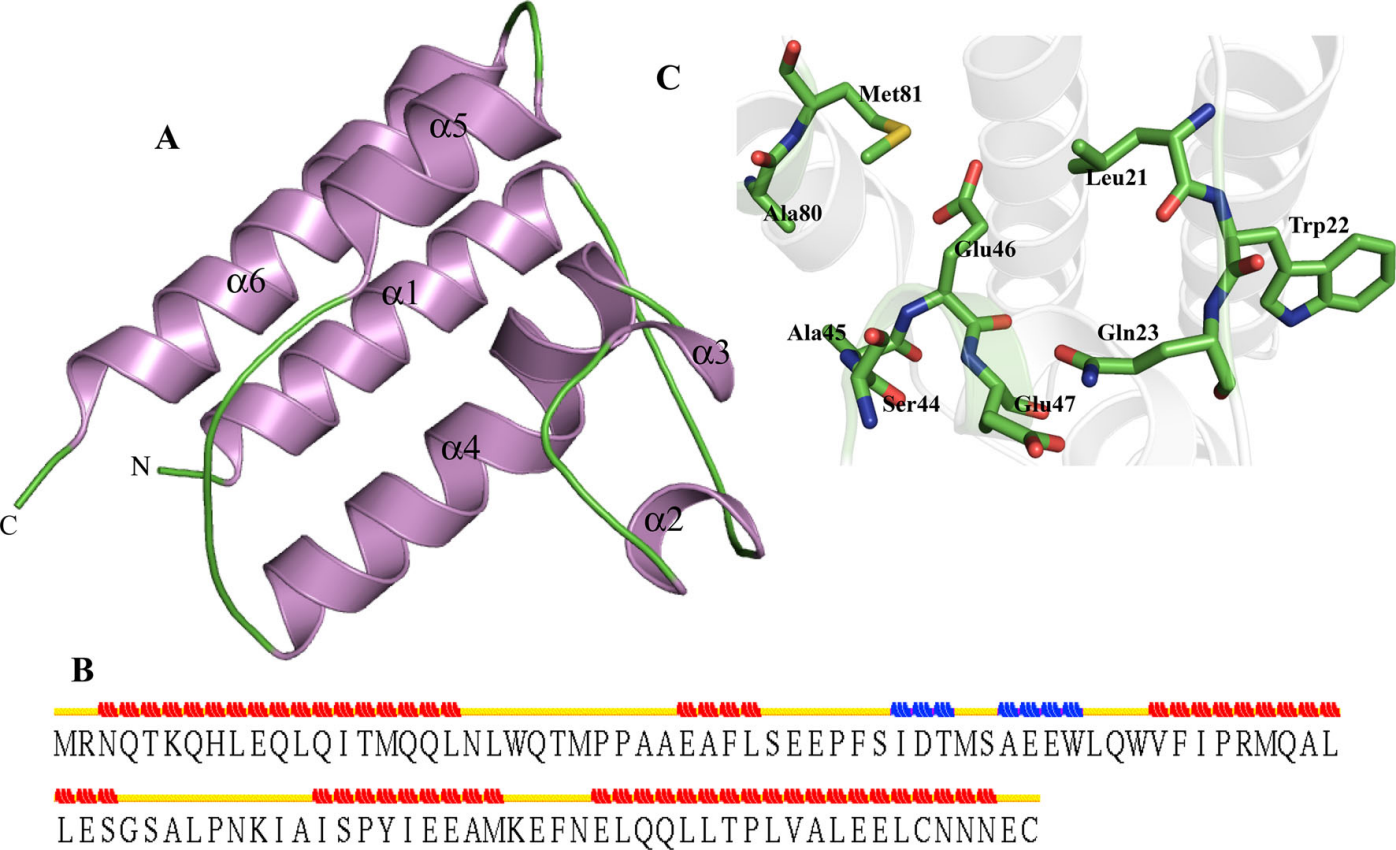
Representation of model structure of HP Q57152. **a** Describing all alpha helix topology in *carton form*. **b** Secondary structure prediction and assignment of STRIDE. **c** Description of active site residues in *stick form*

I-TASSER server was used to predict the structure of HP Q57152 using solution NMR Structure of protein YqcC (PDB ID-2HGK) as a template. Both structures are occupying the similar fold and show close structural similarity (Table 3). We observed 96.9 % of residues are present in the allowed region of Ramachandran plot. Overall structure adopts bromodomain-like fold which has characteristic all α-helix topology (Fig. 11a). Structure of HP Q57152 contains four α-helix and two 3_10_-helices (Fig. 11b). The 3DLigandSite predicts the active of Q57152 contains Leu21, Trp22, Gln23, Ser44, Ala45, Glu46, Glu47, Ala80 and Met81 (Fig. 11c). Further structure analysis shows that the HP may contains β-fructofuranosidase like activity. Function prediction shows variable results indicating HP Q57152 may have multiple functional sites.

### HP P44268

Subcellular localization of HP P44268 suggests that it is localized in cytoplasm with no transmembrane helix and is not involved in any secretory pathways (Table S2). Sequence-based function predictions show that HP P44268 may possess xylose isomerase activity (Table S3 and S4). Uniprot annotation has also indicated that HP P44268 contains a Xyl_isomerase-like TIM barrel domain and belongs to the UPF0276 family that functionally uncharacterised. We further validated our prediction by analyzing the interaction network of P44268 which primarily includes RNA polymerase sigma factor and phosphate transport regulator. We identified three sequence-based motifs in the HP P44268 are 249′-KGTVWD, 99′-CECEGH and 35′-ENWSKM which are helpful in validating the annotation results.

**Fig. 12:**
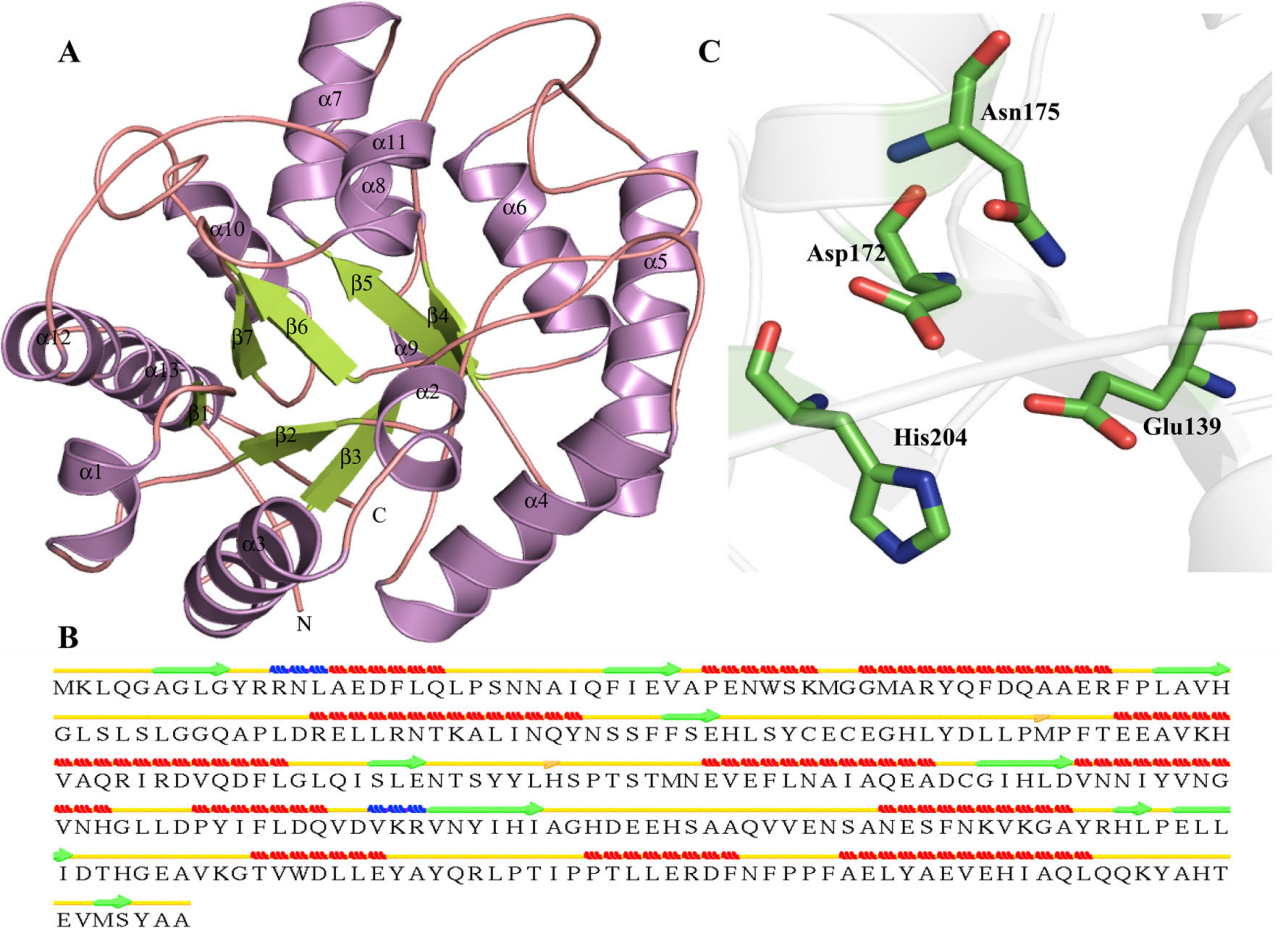
Representation of model structure of HP P44268. **a** Showing characteristic TIM barrel topology. **b** Secondary structure prediction of HP P44268. **c** Representation of active site residues in *stick*

We obtained the DUF692 family protein, a domain of unknown function (PDB ID—3BWW) and L-ribulose 3-epimerase (PDB ID—3VYL) as a templates for homology modeling of HP P44268, which are showing a sequence identity of 73 and 50 %, respectively. The model is showing 98.9 % residues in the allowed region showing high fold similarity with the templates (Table 3). The STRIDE assignment of secondary structure shows that structure of HP P44268 contains 10 β-strands, 12 α-helix and two 3_10_-helices with isolated β-bridges at Met111 and His146 (Fig. 12a). The structure of HP P44268 folds into a TIM alpha/beta-barrel. But TIM barrel of P44268 shows (α/β)_7_ topology instead of (α/β)_8_ with seven β-strands (Fig. 12b). The active site pocket of HP P44268 contains manganese-binding sites at Glu139, Asp172, Asn175, His204 and Glu272 (Fig. 12c). The protein structure is found similar to those of epimerases like L-ribulose 3-epimerase, xylose isomerase domain protein TIM barrel and D-tagatose 3-epimerase (Table S6), while the ProFunc shows that the protein has xylose isomerase like activity. The xylose isomerase is responsible for the isomerization of the pentoses sugars like methyl pentose and even glucose in the bacterial cells (Sanchez and Smiley 1975).

### HP P52606

Sequence analysis of HP P52606 indicates that this protein is localized in the cytoplasm (Table S2). Sequence similarity search and domain analysis show that P51606 may have sedoheptulose 7-phosphate isomerase like activity (Table S3 and S4). Uniprot annotation has also indicated that HP P52606 is involved in carbohydrate metabolic process and acts as isomerase. Furthermore, sequence similarity search suggest that HP P52606 contains a SIS, a phosphosugar-binding domain and belongs to the DiaA subfamily, a DnaA initiator-associating protein DiaA which is required for the timely initiation of chromosomal replication via direct interactions with the DnaA initiator protein, required for DNA replication. We further validated the result using protein–protein interaction analysis that shows that this protein interacted with bifunctional heptose 7-phosphate kinase/heptose 1-phosphate adenyltransferase, antigen, chromosomal replication initiation protein, imidazole glycerol-phosphate dehydratase/histidinol phosphatase and D-heptose 1, 7-bisphosphate phosphatase.

**Fig. 13:**
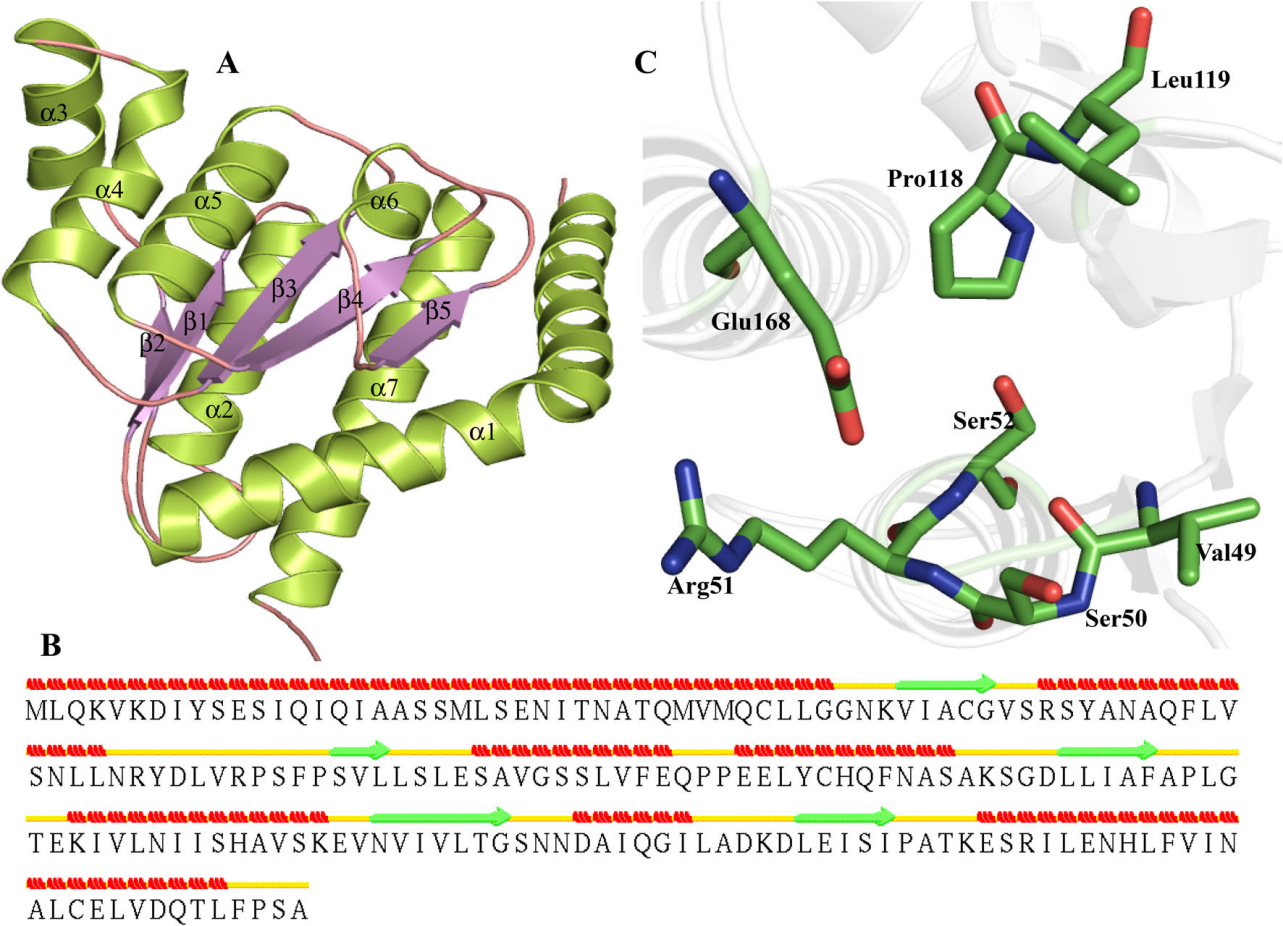
Representation of model structure of HP P52606. **a** Illustration of beta sandwich topology of P52606. **b** Predicted Secondary structural elements in P52606. **c** *Stick* representation of active site residues

Due to moderate similarity (>30 %) of HP P52606 with the crystal structure of *Escherichia coli* DiaA (PDB ID—2YVA) and phosphoheptose isomerases (Table 3), we used MODELLER for structure prediction. The predicted model show high value of TM score (>0.85) indicating a close fold similarity with the templates. Similarly, low RMSD value (<0.550) shows high structure similarity of target and templates. The refined model showed 99.4 % residues in the allowed region of Ramachandran plot. The overall structure of HP P52606 contains seven α-helix and five β-strands (Fig. 13a). The predicted structure of P52606 revealed a SIS domain that contains central five-stranded parallel sheet, flanked by seven α-helices that results in three-layered α-β-α sandwich. The α1, α2 and α7 are present on one side of this sandwich and α3, α4, α5, α6 are present on other side (Fig. 13b). Furthermore, the active site is comprised of Val49, Ser50, Arg51, Ser52, Pro118, Leu119, Glu168 (Fig. 13c). The function of P52606 as a sedoheptulose 7-phosphate isomerase was further validated from the DALI and ProFunc servers (Table S6). The sedoheptulose 7-phosphate isomerase catalyzes the isomerization of D-sedoheptulose 7-phosphate into D-glycero-D-manno-heptose 7-phosphate, the first step in the formation of ADP heptose (Taylor et al. 2008).

## Conclusions

The isomerases have an important role in the virulence of pathogens such as UDP N-acetylgalactosamine 4-epimerase which is found to be involved in the formation of smooth lipopolysaccharide and is essential for the virulence of mesophilic aeromonas hydrophila serotype O34 (Canals et al. 2006). Similarly, UDP-glucose 4-epimerase encoded by galE gene plays an important part in lipopolysaccharide biosynthesis which is one of the main virulence factors of bacterial pathogens (Fry et al. 2000). Our extensive analysis of structures of 13 isomerases characterized from 429 HPs of *H. influenzae* is helpful in identification of putative drug targets for better drug design. HP P71373 annotated as nucleoside-diphosphate-sugar epimerase. Four HPs were identified as a virulent protein which can be used to better understand the virulence mechanism of *H. influenzae* and search for a potential target for therapeutic intervention. Isomerases clearly play a central role in the relationship between bacteria and the host. Our structure-based function elucidation provides an insight how microbes interact with the hosts and will contribute significantly to our understanding of both the isomerase molecule and bacterial pathogenesis in the future.

## Electronic supplementary material

Below is the link to the electronic supplementary material.

Supplementary material 1 (DOCX 17 kb)

Supplementary material 2 (DOC 50 kb)

Supplementary material 3 (DOC 45 kb)

Supplementary material 4 (DOC 46 kb)

Supplementary material 5 (DOC 58 kb)

Supplementary material 6 (DOC 38 kb)
