# Supplementary material for: Structure-based function analysis of putative conserved proteins with isomerase activity from Haemophilus influenzae
Source: 3 Biotech. 2014 Dec 28;5(5):741–63. doi: 10.1007/s13205-014-0274-1 (PMC4569619; doi:10.1007/s13205-014-0274-1)
Supplement: Supplementary file 1 — Supplementary material 1 (DOCX 17 kb) [file 13205_2014_274_MOESM1_ESM.docx]

**Table S1:** List of tools used in this study

| **S. No.** | **Name of Tool** | **Purpose** | **Reference** |
| --- | --- | --- | --- |
|  | PSORTb | Subcellular localization | Yu et al., 2010 |
|  | PSLpred | Subcellular localization | Bhasin et al., 2005 |
|  | CELLO | Subcellular localization | Yu et al., 2006 |
|  | SignalP 4.1 | Signal peptide prediction | Emanuelsson et al., 2007 |
|  | SecretomeP | To identify non-classical secretory pathway | Bendtsen et al., 2005 |
|  | TMHMM | To predict number of α-helix | Krogh et al., 2001 |
|  | HMMTOP | Membrane protein prediction | Tusnady and Simon, 2001 |
|  | BLASTp | To find similar proteins | Altschul et al., 1990 |
|  | SCOP | To find similar structure and fold | Hubbard et al., 1999 |
|  | CATH | To find similar structure and fold | Sillitoe et al., 2013 |
|  | Pfam | To assign superfamily and family annotation to a sequence | Punta et al. |
|  | PANTHER | To find molecular function and biological process categories | Mi et al., 2005 |
|  | SMART | A comprehensive sequence analysis and comparison | Letunic et al. |
|  | SUPERFAMILY | To assign superfamily and family annotation to a sequence | Gough et al., 2001 |
|  | CDART | Performs similarity searches of a sequence based on domain architecture | Geer et al., 2002 |
|  | SYSTERS | Classification of sequences into disjoint protein family clusters and hierarchically into superfamily and subfamily clusters. | Meinel et al., 2005 |
|  | ProtoNet | Offers automatic hierarchical classification of proteins | Rappoport et al. |
|  | SVMProt | Functional Classification of a Protein | Cai et al., 2003 |
|  | MOTIF | Motif-based sequence analysis tools | Kanehisa, 1997 |
|  | InterProScan | For protein signature recognition in a given sequence | Quevillon et al., 2005 |
|  | MEME suite | A collection of tools for the discovery and analysis of sequence motifs | Bailey et al., 2009 |
|  | VICMpred | Virulence factor prediction | Saha and Raghava, 2006 |
|  | STRING (version–9.05) | To find functional partner | Szklarczyk et al., 2010 |
|  | MODELLER | To model the protein structure | Eswar et al., 2006 |
|  | I-TASSER | To model the protein structure | Roy et al., 2010 |
|  | ROBETTA server | To model the protein structure | Kim et al., 2004 |
|  | HHpred | For fold recognition | Soding et al., 2005 |
|  | Deepview | For energy minimization of model | Kaplan and Littlejohn, 2001 |
|  | PROCHECK | To validate the model | Laskowski et al., 1996 |
|  | PyMOL | To visualize the protein structure | DeLano, 2002 |
|  | COACH | To predict catalytic and ligand-binding residues | Yang et al., 2013 |
|  | COFACTOR | To predict catalytic and ligand-binding residues | Roy et al., 2012 |
|  | 3DLigandSite | To predict catalytic and ligand-binding residues | Wass et al., 2010 |
|  | TM-SITE | To predict catalytic and ligand-binding residues | Yang et al., 2013 |
|  | S-SITE | To predict catalytic and ligand-binding residues | Yang et al., 2013 |
|  | POCASA | To find ligand binding pocket | Yu et al., 2010 |
|  | Pocket-Finder | To find ligand binding pocket | Laurie and Jackson, 2005 |
|  | ProFunc | To predict the function of protein | Laskowski et al., 2005 |
|  | DALI server | To find structurally similar proteins in the PDB | Holm and Rosenstrom |
|  | STRIDE web server | To predict secondary structure | Heinig and Frishman, 2004 |
