# Supplementary material for: Structure-based function analysis of putative conserved proteins with isomerase activity from Haemophilus influenzae
Source: 3 Biotech. 2014 Dec 28;5(5):741–63. doi: 10.1007/s13205-014-0274-1 (PMC4569619; doi:10.1007/s13205-014-0274-1)
Supplement: Supplementary file 2 — Supplementary material 2 (DOC 50 kb) [file 13205_2014_274_MOESM2_ESM.doc]

| **S.No.**  **Table S2:** Prediction of sub-cellular localization of HP with isomerase activity in *H. influenzae* strain Rd KW20 | **UNIPROT ID** | **PSORT B** | **PSLpred** | **CELLO** | **Signal peptide** | **SecretomeP**  (Secretion) | **HMMtop** | **TMHMM** |
| --- | --- | --- | --- | --- | --- | --- | --- | --- |
|  | P44506 | Cytoplasmic | Inner membrane protein | Cytoplasmic | No | No | No TM helix | No TM helix |
|  | P44641 | Cytoplasmic | Cytoplasmic protein | Cytoplasmic | No | No | No TM helix | No TM helix |
|  | P46494 | Unknown | Cytoplasmic protein | Periplasmic | No | Yes | No TM helix | No TM helix |
|  | P44827 | Cytoplasmic | Cytoplasmic | Cytoplasmic | No | No | No TM helix | No TM helix |
|  | Q57151 | Cytoplasmic | Cytoplasmic protein | Cytoplasmic | No | No | No TM helix | No TM helix |
|  | P44094 | Cytoplasmic | Cytoplasmic protein | Cytoplasmic | No | No | No TM helix | No TM helix |
|  | P45104 | Cytoplasmic | Cytoplasmic protein | Cytoplasmic | No | Yes | No TM helix | No TM helix |
|  | P71373 | Cytoplasmic | Cytoplasmic protein | Cytoplasmic | No | No | No TM helix | No TM helix |
|  | P44160 | Cytoplasmic | Cytoplasmic protein | Cytoplasmic | No | Yes | No TM helix | No TM helix |
|  | O86237 | Cytoplasmic | Cytoplasmic protein | Cytoplasmic | No | No | No TM helix | No TM helix |
|  | Q57152 | Unknown | Periplasmic protein | Cytoplasmic | No | No | No TM helix | No TM helix |
|  | P44268 | Cytoplasmic | Cytoplasmic protein | Cytoplasmic | No | No | No TM helix | No TM helix |
|  | P52606 | Cytoplasmic | Cytoplasmic protein | Cytoplasmic | No | No | No TM helix | No TM helix |
