# Supplementary material for: Structure-based function analysis of putative conserved proteins with isomerase activity from Haemophilus influenzae
Source: 3 Biotech. 2014 Dec 28;5(5):741–63. doi: 10.1007/s13205-014-0274-1 (PMC4569619; doi:10.1007/s13205-014-0274-1)
Supplement: Supplementary file 3 — Supplementary material 3 (DOC 45 kb) [file 13205_2014_274_MOESM3_ESM.doc]

| **S.NO**  **Table S3**: List of sequence based predicted function of HP with isomerase activity in *H. influenzae* strain Rd KW20 | **UNIPROT ID** | **BLAST** | **Predicted functional partner**  **(STRING)** | **SMART** | **INTERPROSCAN** | **MOTIF** | **Virulentpred** | **VICMpred** |
| --- | --- | --- | --- | --- | --- | --- | --- | --- |
|  |  |  |  |  | **MOTIF FOUND** | | **Virulence factors** | |
| 1 | P44506 | putative pyridoxal phosphate-dependent enzyme, YBL036C type | Holliday junction resolvase-like protein | Alanine racemase | Alanine racemase | Proline synthetase  (PRODOM) | Yes | Metabolism molecule |
| 2 | P44641 | lysine 2,3-aminomutase | elongation factor P | Radical SAM , L-lysine 2,3-aminomutase (SCOP) | Lysine 2,3-aminomutase like | L-lysine 2,3-aminomutase  (PRODOM) | No | Cellular process |
| 3 | P46494 | DNA topoisomerase | DNA topoisomerase III | Topoisomerase DNA binding C4 zinc finger | DNA Topoisomerase ,type IA ,zn finger | Topoisomerase I, zinc-ribbon-like  (BLOCKS) | No | Cellular process |
| 4 | P44827 | ribosomal large subunit pseudouridine synthase E | lipoprotein E | RNA pseudouridylate synthase | Pseudouridine synthase, RsuA/RluB/E/F | Pseudouridine synthase, Rsu  (BLOCKS) | No | Metabolism molecule |
| 5 | Q57151 | hydroxypyruvate isomerase | 3-hydroxyisobutyrate dehydrogenase | Xylose isomerase-like TIM barrel | Xylose isomerase-like, TIM barrel domain | Hydroxypyruvate isomerase  (PRODOM) | No | Cellular process |
| 6 | P44094 | NAD-dependent epimerase/dehydratase | 3-hydroxyisobutyrate dehydrogenase | Nucleoside-diphosphate-sugar epimerase | NAD-dependent epimerase/dehydratase | Nucleoside-diphosphate-sugar epimerase  (PRODOM) | No | Cellular process |
| 7 | P45104 | ribosomal large subunit pseudouridine synthase B | 23S rRNA pseudouridine synthase D | Ribosomal small subunit pseudouridine synthase | Pseudouridine synthase, RsuA/RluB/E/F | Pseudouridine synthase, Rsu  (BLOCKS) | No | Cellular process |
| 8 | P71373 | Amidophosphoribosyltransferase (Epimerase) | arginine repressor | NAD dependent epimerase/dehydratase | NAD dependent epimerase/dehydratase | NAD-dependent epimerase/dehydratase  (BLOCKS) | Yes | Virulence factor |
| 9 | P44160 | glucose-6-phosphate 1-epimerase | glucose-6-phosphate isomerase | Aldose 1-epimerase | Galactose mutarotase-like | Aldose 1-epimerase  (BLOCKS) | Yes | Cellular process |
| 10 | O86237 | anthranilate phosphoribosyltransferase | anthranilate synthase component II | Tautomerase/MIF | Tautomerase/MIF | 4-oxalocrotonate tautomerase  (BLOCKS) | No | Cellular process |
| 11 | Q57152 | RNA pseudouridine synthase C -like domain | penicillin-binding protein 1B | Dihydropteroate synthase-like | Protein of unknown function UCP006257 | Pseudouridine synthase C  (PRODOM) | Yes | Cellular process |
| 12 | P44268 | No result | RNA polymerase sigma factor | Xylose isomerase-like / metal binding proteins | Xylose isomerase-like, TIM barrel domain | 56kDa selenium binding  (BLOCKS) | No | Cellular process |
| 13 | P52606 | DnaA initiator-associating factor for replication initiation | bifunctional heptose 7-phosphate kinase/heptose 1-phosphate adenyltransferase | DnaA initiator-associating protein DiaA like | Sedoheptulose 7-phosphate isomerase / dnaa initiator-associating factor for replication initiation | SIS domain profile(Sugar ISomerase)  (PROSITE PROFILE) | No | Metabolism molecule |
