# Supplementary material for: Structure-based function analysis of putative conserved proteins with isomerase activity from Haemophilus influenzae
Source: 3 Biotech. 2014 Dec 28;5(5):741–63. doi: 10.1007/s13205-014-0274-1 (PMC4569619; doi:10.1007/s13205-014-0274-1)
Supplement: Supplementary file 4 — Supplementary material 4 (DOC 46 kb) [file 13205_2014_274_MOESM4_ESM.doc]

| **S.NO**  **Table S4**: List of functionally annotated domain of 13 HPs with isomerase activity from *H. influenzae* strain Rd KW20 | **UNIPROT ID** | **CATH** | **SUPERFAMILY** | **PANTHER families**  **(subfamily/family /superfamily )** | **Pfam**  **(family/Domain)** | **SYSTERS**  **(cluster)** | **CDART**  **@NCBI** | **SVMProt (family)** | **ProtoNet** |
| --- | --- | --- | --- | --- | --- | --- | --- | --- | --- |
| 1 | P44506 | Alanine racemase -like domain family | "Hypothetical" protein ybl036c family  (PLP-binding barrel superfamily) | Proline synthetase co-transcribed bacterial homolog protein family | Ala_racemase_N family  (Alanine racemase, N-terminal domain) | Cluster-140661  (Putative Prolinesynthetase associated protein) | PLPDE_III superfamily | Metal binding | Cluster 4106519  Predicted pyridoxal phosphate-dependent enzyme, YBL036C type |
| 2 | P44641 | L-lysine 2,3-aminomutase -like domain family | MoCo biosynthesis proteins family | lysine 2,3-aminomutase like family | Fer4_14 family | Cluster- 133235  (L-lysine 2,3-aminomutase) | Radical_SAM Superfamily | Iron-binding | Cluster 4031921  Protein of unknown function DUF160 |
| 3 | P46494 | DNA topoisomerase -like domain 1/2/3 family | PhnA zinc-binding domain family/  Prokaryotic DNA topoisomerase I, a C-terminal fragment family | Prokaryotic dna topoisomerase family | zf-C4_Topoisom family | Cluster- 144826  (DNA topoisomerase I) | TOPRIM Superfamily | Zinc-binding | Cluster 4554047  Prokaryotic type I DNA topoisomerase |
| 4 | P44827 | No result | Pseudouridine synthase RsuA/RluD family | Ribosomal large subunit pseudouridine synthase bfamily | PseudoU_synth_2 family | Cluster- 141200  (Ribosomal large subunit pseudouridine synthase B) | PseudoU_synth Superfamily | rRNA-binding Proteins | Cluster 4152309  Pseudouridine synthase, RsuA and RluB/E/F |
| 5 | Q57151 | Hydroxypyruvate isomerase -like domain family | Xylose isomerase-like superfamily | Hydroxypyruvate isomerase family | AP_endonuc_2 family | Cluster – 148356  (Hydroxypyruvate isomerase) | AP2Ec Superfamily | EC 2.4.-.-: Transferases - Glycosyltransferases | Cluster4151021  Hydroxypyruvate isomerase |
| 6 | P44094 | CDP-abequose synthase -like domain family/  NAD dependent epimerase/dehydratase family protein -like domain | Tyrosine-dependent oxidoreductases family | NAD dependent epimerase/dehydratase family | Epimerase family | Cluster – 142884  (Nucleoside-diphosphate-sugar epimerase) | NADB_Rossmann Superfamily | EC 1.1.-.-: Oxidoreductases - Acting on the CH-OH group of donors | Cluster4107863  NAD-dependent epimerase/dehydratase |
| 7 | P45104 | 30S ribosomal protein S4 -like domain family | Pseudouridine synthase RsuA/RluD family | RIbosomal large subunit pseudouridine synthase b family | S4 family | Cluster – 141200  (Ribosomal large subunit pseudouridine synthase B) | PseudoU_synth Superfamily | RNA-binding Proteins | Cluster4152309  Pseudouridine synthase, RsuA and RluB/E/F |
| 8 | P71373 | Putative uncharacterized protein -like domain family | Tyrosine-dependent oxidoreductases family | Sugar nucleotide epimerase related family | Epimerase family | Cluster – 146743  (Cell-division inhibitor) | NADB_Rossmann Superfamily | EC 2.7.-.-: Transferases - Transferring Phosphorus-Containing Groups | Cluster3947300  NAD(P)-binding Rossmann-like Domain |
| 9 | P44160 | Aldose 1-epimerase family protein -like domain family | Galactose mutarotase-like superfamily | Apospory-associated protein c-related family | Aldose_epim family | Cluster – 112182  (kinase like protein) | Aldose_epim Superfamily | No result | Cluster4158146  Aldose 1-epimerase |
| 10 | O86237 | Macrophage migration inhibitory factor -like domain family | Tautomerase/MIF superfamily | No result | Tautomerase_2 family | Cluster – 156324  (4-oxalocrotonate tautomerase) | No result | EC 3.1.-.-: Hydrolases - Acting on Ester Bonds | Cluster4016578  Macrophage Migration Inhibitory Factor |
| 11 | Q57152 | tRNA pseudouridine synthase C -like domain family | YqcC-like family | No result | DUF446 family  (tRNA pseudouridine synthase C) | Cluster – 111794  (yqcC protein) | DUF446 Superfamily | No result | Cluster4058700  Protein of unknown function DUF446 |
| 12 | P44268 | UPF0276 protein MCA3108 -like domain family | Xylose isomerase-like superfamily | No result | DUF692 family | Cluster – 133486  (Hypothetical protein) | DUF692 Superfamily | Manganese-binding | Cluster41293114  Protein of unknown function DUF692  (Xylose isomerase-like) |
| 13 | P52606 | Glucose-6-phosphate isomerase -like domain ½ family | mono-SIS domain family | Sedoheptulose 7-phosphate isomerase / dnaa initiator-associating factor for replication initiation family | SIS_2 family  (Sugar ISomerase) | Cluster – 147050  (Phosphoheptose isomerase (EC 5.-.-.-)) | SIS Superfamily  (Sugar ISomerase) | Manganese-binding | Cluster3887230  Interconverting aldoses and ketoses, and related compounds |
