# Supplementary material for: Structure-based function analysis of putative conserved proteins with isomerase activity from Haemophilus influenzae
Source: 3 Biotech. 2014 Dec 28;5(5):741–63. doi: 10.1007/s13205-014-0274-1 (PMC4569619; doi:10.1007/s13205-014-0274-1)
Supplement: Supplementary file 5 — Supplementary material 5 (DOC 58 kb) [file 13205_2014_274_MOESM5_ESM.doc]

| **S. No.**  **Table S5:** List predicted binding site residues of HPs with isomerase activity from *H. influenzae* | **Uniprot ID** | **Template structure comparison** | **Firestar** | **3DLigandSite** | **COACH** | **COFACTOR** | **TM-SITE** | **S-SITE** |
| --- | --- | --- | --- | --- | --- | --- | --- | --- |
|  | P44506 | Active site  K35- PLP binding site  (1W8G and 1VFS) | | SITE 1 Compound_name: PYRIDOXAL-5-PHOSPHATE | | --- | | SITE 1 Residues resume: V(33); K(35); N(56); I(80); M(162); I(164); M(204); T(205); R(220); I(221); G(222); T(223); R(229);   | SITE 2 Compound_name: ISOCITRIC ACID | | --- | | SITE 2 Residues resume: Q(84); S(85); R(103); K(105); I(106); | | | PLP binding site-  VAL33, LYS35, ASN56, ILE80, ILE164, ET204, THR205, RG220, ILE221, LY222, THR223 | PLP binding site residues-  33,35,56,80,162,205,220,222,223 | PLP binding site residues-  33,35,56,80,164,220,222,223 | PLP binding site residues-  33,35,54,80,82,163,204,206,220,222,223 | PLP binding site residues-  33,35,56,80,162,164,204,205,220,221,222,223,229 |
|  | P44641 | Active site residues-  C121- Iron-sulfur binding (SAM)  C125- Iron-sulfur binding  C128- Iron-sulfur binding | | SITE 1 Compound_name: IRON/SULFUR CLUSTER |  | | --- | --- | | SITE 1 Residues resume: C(121); N(124); C(125); Y(127); C(128); F(129); S(164); G(165); G(166); D(167); P(168); H(195); T(196); R(197); H(226); Q(254); V(256); H(286); D(289); A(294);   | SITE 2 Compound_name: PYRIDOXAL-5-PHOSPHATE | | --- | | SITE 2 Residues resume: L(106); K(108); Y(109); R(112); L(114); I(162); R(193); Y(283); Y(284); K(333);   | SITE 3 Compound_name: LYSINE | | --- | | SITE 3 Residues resume: L(94); L(114); R(130); I(162); S(164); H(286); D(289); | | | | | N-acetyl-D-glucosamine (NAG)binding site-  VAL123, CYS125, TYR127, CYS128, PHE129, ARG130, ARG131, HIS132, PRO134, TYR135, GLY166, ASP167, HIS195, ARG197, HIS226, VAL291 | SAM binding site residues-  127,128,129,130,164,165,166,167,195,196,226,228,256,286,287,288,289 | SAM binding site residues-  127,129,130,164,165,166,195,226,254,256,286,287,288,289,294 | SAM binding site residues-  127,128,129,130,164,165,166,167,195,196,226,228,256,286,287,288,289 | UUU binding site residues-  121,125,127,128,129,130,164,165,166,195,197,226,254,256,286,287,288,289,294 |
|  | P46494 | Cys15- zinc binding site  Cys18- Zinc binding site  Cys35- Zinc binding site  Cys41 – Zinc Binding site  Cys104- Zinc binding site  Cys107 – Zinc binding site  Cys145- Zinc binding site  Cys148- Zinc binding site | SITE1 Compound_name:  FE (III) ION  SITE1Residues resume: C(15); C(18); C(62); C(65)  SITE2 Compound_name:  FE (III) ION  SITE2 Residues resume: C(145); C(148); C(167) | ZN binding site-CYS145, THR147, CYS148, CYS167, CYS172, HIS 174 | ZN binding site-  104,107,167,172 | ZN binding site-  15,18,35,41 | ZN binding site-  104,107,167,172,174 | ZN binding site-  104,107,108,109,126,128,132,134 |
|  | P44827 | Asp91 – nucleic acid binding site  (active site) | SITE1 Compound_name:  (5S,6R)-5-Fluoro-6-hydroxy-pseudouridine-5-monophosphate  SITE1Residues resume: G(88); L(90); D(91); K(119); Y(121); | Cu and Zinc binding site –  ILE128 , PRO129, GLU130, ASP133, PHE200 | FOU binding site-  90,91,94,121,191,192,205 | Nucleic acid binding site-  10,11,12,15,32,33,63,66,67,83,85,88,89,91,92,94,104,107,108,111,112,121,186,187,188,189,190,191,192,205,207 | FOU binding site- 91,94,121,189,190,191,205 | URA binding site-89,90,91,94,121,183,188,189,190,191,192,205 |
|  | Q57151 | Active site from template-  Glu143, Asp 178, Gln204, Glu240 | SITE1 Compound_name:  MANGANESE (II) ION  SITE1Residues resume: E(143); D(178); Q(204); R(211); E(240) | MG binding site- ASP178, GLN204, GLU240 | SOR binding site-  35,60,103,105,143,145,178,181,204,240 | MG binding site-143,178,204,211,240 | Mn binding site-35,105,143,145,178,181,204,240 | Zn binding site-7,59,60,61,104,105,110,143,151,178,181,204,211,240,242 |
|  | P44094 | NAD binding site-  Ser80, Tyr143, Lys147  Active site-  Tyr143, Ser185, Arg210 | SITE1 Compound_name:  NICOTINAMIDE-ADENINE-DINUCLEOTIDE  SITE1Residues resume:  G(7); Q(9); G(10); F(11); L(12); D(35); N(54); L(55); L(75); A(76); A(77); I(78); T(93); S(117); S(118); S(119); Y(143); K(147); P(177); G(178)  SITE2 Compound_name:  PYRUVIC ACID  SITE 2 Residues resume:  V(79); S(80); S(119); L(120); Y(143); L(170); P(171); T(172); S(185); W(283); | NDP binding site- THR6, GLY7, GLY8, GLN9, GLY10, PHE11, LEU12, VAL37, MET53, ASN54, LEU55, LEU75, ALA76, ALA77, ILE78, THR93, SER117, SER118, SER119, TYR143, LYS147, ARG169, LEU170, PRO171, THR172, ILE173, ARG176 | NAD binding site-7,9,10,11,12,35,36,37,38,39,40,53,54,55,75,76,77,93,117,118,119,143,147,170,171,172,173 | APR binding site-7,9,10,11,12,35,36,53,54,55,75,76,77,89,93,116,143,147 | NAD binding site-7,9,10,11,12,35,36,37,53,54,55,75,76,77,78,93,117,118,119,143,147,170,171,172,173,178,179,182,183 | NAD binding site-7,9,10,11,12,35,36,37,38,39,40,53,54,55,75,76,77,79,93,117,118,119,143,147,170,171,172,173 |
|  | P45104 | Active site –  Asp183 | SITE1 Compound_name: (5S,6R)-5-FLUORO-6-HYDROXY-PSEUDOURIDINE-5-MONOPHOSPHATE  SITE1Residues resume:  G(180); L(182); D(183); Y(213); R(270); L(283); R(285); | NI binding site  PHE247, GLY249, GLY250, ASN254, GLN255, TYR257 | FOU binding site-  183,186,213,267,268,269,283 | Nuc.Acid binding site-  77,79,80,83,89,90,91,94,110,111,112,113,114,152,153,154,155,156,177,180,181,182,183,184,186,196,197,199,200,203,204,205,206,208,211,213,264,266,267,268,269,283,285,288 | FOU binding site-  183,186,213,267,268,269,283 | URA binding site –  181,182,183,186,213,261,266,267,268,269,270,283 |
|  | P71373 | NADP binding region-  Arg88  Leu66, Ala67, Gly68, Glu69 | SITE1 Compound_name: NICOTINAMIDE-ADENINE-DINUCLEOTIDE  SITE1Residues resume:  G(7); T(9); G(10); L(11); I(12); T(31); R(32); L(66); A(67); G(68); S(110); S(112); G(160); V(162); | NDP binding site-  GLY7, THR9, GLY10, LEU11, ILE12, GLY13, LEU30, THR31, ARG32, LEU66, ALA67, GLY68, GLU69, ARG88, LEU91, GLY111, SER112, ALA113, TRP143, ARG158, THR159, GLY160, MET161, VAL162, MET200 | NDP binding site-  7,9,10,11,12,31,47,66,67,68,70,111,112,113,140,159,160,161,162 | NDP binding site-  7,9,10,11,12,31,32,66,67,68,69,70,87,88,111,112,143,159,160,162 | NDP binding site-  7,9,10,11,12,31,32,66,67,68,70,91,111,112,140,159,160,162 | NDP binding site-  7,9,10,11,12,31,32,66,67,68,69,70,83,87,88,111,112,143,159,160,162 |
|  | P44160 | Active site-  His151, Glu249 | SITE1 Compound_name: BETA-D-GALACTOSE  SITE1Residues resume:  H(88); H(150); Y(152); D(192); E(248); | GAL binding site-  ARG71, PHE81 , HIS89, HIS151, TYR153, ASP193, TRP227, GLU249 | GAL binding site-  70,81,87,88,150,152,192,224,226,248 | TA6 binding site-  70,87,88,92,150,192,224,226,248 | GAL binding site-  70,81,87,88,150,152,192,224,226,248 | TA6 binding site-  70,80,87,88,92,150,174,192,224,226,232,236,248 |
|  | O86237 | Asp48, Lys85, Glu103 | No result | SFD binding site-  PHE5, GLU63, ASN65, LYS102 | FUN binding site-  1,32,36,67,109,111,116 | GLU binding site-  36,37,109,111,112 | PR6 binding site-  1,32,36,67,109,111,116 | PR6 binding site-  36,66,67,70,104,109,118,120 |
|  | Q57152 | No result | No result | MG binding site-  LEU21, TRP22, GLN23, SER44, ALA45, GLU46, GLU47, ALA80, MET81 | HEA binding site-  90,91 | RED binding site-  46,53,76,77 | WJ1 binding site-  12,49,71,73,94,97,98,101 | MG binding site-  52,89,90,91,94,97 |
|  | P44268 | Manganese binding site -  His204 | SITE1 Compound_name: MANGANESE (II) ION  SITE1Residues resume:  E(139); D(172); H(204); E(272);  SITE2 Compound_name:  FE (III) ION  SITE1Residues resume:  H(60); H(95); E(139); | MG binding site-  GLU139, ASP172, ASN175, HIS204, GLU272 | MG binding site-  139,172,204,272 | FE binding site-  60,95,139 | MG binding site-  33,95,139,172,175,204,272 | FE binding site-  60,95,139,172,204,272 |
|  | P52606 | Active site-  Ser52, Arg71, Pro72, Phe190, Pro191 | SITE1 Compound_name: SULFATE ION  SITE1Residues resume:  G(48); V(49); S(50); R(51); S(52); | SUC binding site-  VAL49, SER50 , ARG51, SER52, PRO118, LEU119, GLU168 | M7P binding site-  49,50,51,117,118,119,122,168,172 | BMX binding site-  49,51,52,117,118,119,122 | I22 binding site-  58,62,180 | M7P binding site-  58,62,65,66,180 |
