# Supplementary material for: Structure-based function analysis of putative conserved proteins with isomerase activity from Haemophilus influenzae
Source: 3 Biotech. 2014 Dec 28;5(5):741–63. doi: 10.1007/s13205-014-0274-1 (PMC4569619; doi:10.1007/s13205-014-0274-1)
Supplement: Supplementary file 6 — Supplementary material 6 (DOC 38 kb) [file 13205_2014_274_MOESM6_ESM.doc]

**Table S6:** List of predicted structure analysis results of HP with isomerase activity *H. influenzae* strain Rd KW20

| **S. NO.** | **UNIPROT ID** | **^b^ ProFunc** | **^c^ DALI server** |
| --- | --- | --- | --- |
|  | P44506 | Pyridoxal 5'-phosphate-dependent enzyme | Alanine racemase |
|  | P44641 | lysine-2, 3-aminomutase | lysine-2, 3-aminomutase |
|  | P46494 | zf-C4_Topoisom | DNA topoisomerase I |
|  | P44827 | Pseudouridine synthase | Pseudouridine synthase |
|  | Q57151 | hydroxypyruvate isomerase/ xylose isomerase | D-tagatose 3-epimerase |
|  | P44094 | NAD dependent epimerase/dehydratase | NDP-sugar epimerase/ L-threonine dehydrogenase/ CDP-tyvelose-2-epimerase/ UDP-glucose 4-epimerase |
|  | P45104 | ribosomal large subunit pseudouridine synthase B | ribosomal large subunit pseudouridine synthase B and ribosomal large subunit pseudouridine synthase F |
|  | P71373 | Sugar nucleotide epimerase related | UDP-glucose 4-epimerase |
|  | P44160 | Aldose 1-epimerase | Aldose 1-epimerase |
|  | O86237 | Tautomerase/MIF | malonate semialdehyde decarboxylase /putative tautomerase |
|  | Q57152 | No result | beta-fructofuranosidase/ invertase inhibitor |
|  | P44268 | Xylose isomerase-like | L-ribulose 3-epimerase/xylose isomerase domain protein TIM barrel/ D-tagatose 3-epimerase |
|  | P52606 | Sedoheptulose 7-phosphate isomerase / dnaa initiator-associating factor for replication initiation | Phosphoheptose isomerase |

^b^. <http://www.ebi.ac.uk/thornton-srv/databases/ProFunc/>

^c^. <http://ekhidna.biocenter.helsinki.fi/dali_lite/start>
